# Infection Biomarkers in Children with Chemotherapy-Induced Severe Neutropenia

**DOI:** 10.3390/cancers17132227

**Published:** 2025-07-02

**Authors:** Wioletta Bal, Zuzanna Piasecka, Klaudia Szuler, Radosław Chaber

**Affiliations:** 1 Department of Pediatrics, Faculty of Medicine, University of Rzeszow, 35-310 Rzeszow, Poland; wbal@ur.edu.pl (W.B.); zuz.pia@wp.pl (Z.P.); kszuler@ur.edu.pl (K.S.); 2 Clinic of Pediatric Oncology and Hematology, State Hospital 2, 35-301 Rzeszow, Poland

**Keywords:** biomarker, febrile neutropenia, children, sepsis, bacteriemia

## Abstract

Background/Objectives: Febrile neutropenia is a frequent and potentially life-threatening complication in pediatric oncology patients receiving chemotherapy. Due to profound immunosuppression, early diagnosis of infections remains a major clinical challenge. This review evaluates the diagnostic and prognostic utility of infection biomarkers in children with chemotherapy-induced severe neutropenia. Methods: We reviewed clinical studies that assessed the diagnostic performance of inflammatory biomarkers—including C-reactive protein (CRP), procalcitonin (PCT), interleukins (IL-6, IL-8, IL-10), and others—in pediatric febrile neutropenia. The review includes data on sensitivity, specificity, predictive value, and clinical applications. Results: CRP remains a common but nonspecific marker, often insufficient for early stratification. PCT showed consistently high negative predictive value and early responsiveness to bacterial infections. IL-6 and IL-10 demonstrated strong early diagnostic accuracy in the early phase (AUC > 0.80 in multiple studies) and were particularly useful in predicting septic shock when combined. IL-8, while less specific, may help rule out infection when levels are low. Emerging biomarkers such as presepsin, MR-proADM, and PSP showed promising diagnostic performance. Presepsin achieved near-perfect accuracy in some cohorts (AUC up to 0.996), outperforming CRP and PCT, though its ability to discriminate bacteremia at fever onset varied. MR-proADM demonstrated consistent AUCs above 0.75 and may support early sepsis identification. PSP was associated with significantly elevated levels in sepsis. Additional novel markers—including sTNFR-II, sIL-2R, IP-10, Flt-3L, MCP-1-a, and MBL—showed encouraging diagnostic profiles in individual studies, particularly due to high specificity, but require external validation. G-CSF also emerged as a promising candidate in multimarker models. In contrast, TNF-α and IL-1β displayed limited utility as standalone indicators. Conclusions: Biomarkers such as PCT, IL-6, Il-8, and IL-10 offer valuable tools for early infection detection and risk stratification in pediatric febrile neutropenia. Emerging markers—including presepsin, MR-proADM, and PSP—further enhance diagnostic precision and may support early identification of sepsis. Multimarker strategies, particularly those incorporating presepsin, IL-10, or MR-proADM, show potential to improve diagnostic performance beyond conventional markers. Further prospective validation is needed to optimize clinical implementation and guide personalized treatment decisions.

## Simple Summary

Children undergoing chemotherapy for cancer often experience a serious drop in white blood cells, which makes them highly vulnerable to infections. These infections are difficult to detect early because the immune system’s usual warning signals may not appear. Our review explores how different biological markers in the blood—such as certain proteins and immune signals—can help detect infections earlier and more accurately in these children. By improving the understanding and application of these markers in routine care, unnecessary treatments may be reduced, outcomes improved, and children helped to recover more safely during cancer therapy.

## 1. Introduction

Chemotherapy-induced toxicities, particularly myelosuppression and mucositis, constitute major drivers of infection risk in pediatric oncology. Neutropenia, a frequent complication of cancer therapy, markedly increases susceptibility to infections, with the nadir risk occurring when the absolute neutrophil count (ANC) falls below 100 cells/mm^3^ [1]. Chemotherapy further amplifies this vulnerability by impairing neutrophil chemotaxis and phagocytic function. Endogenous microbiota, translocating across mucosal barriers damaged by cytotoxic agents in the oral and gastrointestinal epithelium, represent the primary infection source. This risk is compounded by indwelling vascular or urinary catheters. The temporal progression of mucositis mirrors neutropenia dynamics, emerging at the neutrophil nadir and resolving alongside the count recovery. Collectively, impaired host defenses, neutropenia, mucosal disruption, and catheter use create a multifactorial risk profile for bacterial, fungal, and viral infections [2].

These overlapping risk factors significantly increase the susceptibility of children to life-threatening infections, emphasizing the critical need for early diagnostic precision and accurate prognostic assessment to improve clinical outcomes. Cytokines—including interleukins (ILs), tumor necrosis factors (TNFs), and interferons (IFNs)—play central roles in coordinating immune responses and modulating inflammatory cascades during infectious episodes. Recent evidence has identified IL-6, IL-8, and IL-10, alongside acute-phase reactants such as C-reactive protein (CRP) and procalcitonin (PCT), as promising biomarkers for early infection detection in neutropenic patients, supporting timely and targeted therapeutic interventions [3,4].

However, translating cytokine biomarkers into clinical practice faces significant hurdles in pediatric neutropenia. Profound chemotherapy-induced leukopenia alters cytokine concentrations and functional responses, while confounding variables such as infection etiology, comorbidities, and malignancy biology complicate interpretation, necessitating rigorous standardization for reliable implementation [5]. Deciphering cytokine interaction networks and pathogen-specific signaling patterns could revolutionize risk stratification and enable personalized therapeutic approaches, potentially improving survival and the quality of life in this vulnerable population.

The aim of this review is to synthesize current evidence on the diagnostic and prognostic utility of various biomarkers for identifying severe infections in children with cancer and neutropenia. Integrating these biomarkers into routine clinical practice holds the potential to enhance early risk stratification, guide therapeutic decisions, and ultimately improve outcomes in this high-risk pediatric population.

## 2. Diagnostic Biomarkers of Infectious Etiology in Pediatric Febrile Neutropenia

The key studies evaluating diagnostic biomarkers in febrile neutropenic children are summarized in Table 1. In multiple independent cohorts, IL-6, IL-10, IL-8, PCT, and CRP have demonstrated some diagnostic relevance among the investigated molecules. While C-reactive protein remains widely used due to its accessibility and affordability, newer markers such as PCT and interleukins (IL-6, IL-8, IL-10) have demonstrated promising diagnostic and prognostic performance. Combinatorial biomarker panels offer even greater accuracy, especially for early infection detection and risk stratification in pediatric febrile neutropenia (FN). Below, key biomarkers are discussed according to their utility in distinguishing infectious from non-infectious fever, identifying specific infectious agents (Gram-positive bacterial (GPB), Gram-negative bacterial (GNB), viral), and informing clinical decisions including risk assessment and antimicrobial stewardship.

### 2.1. C-Reactive Protein

C-reactive protein is a classical acute-phase protein and a key component of the early immune response. Synthesized primarily by hepatocytes in response to interleukin-6 and other pro-inflammatory cytokines, CRP levels can rise rapidly within hours of infection or tissue injury, often reaching peak concentrations within 48 h [51]. This makes it one of the most widely used biomarkers in the clinical setting for detecting and monitoring inflammation. Despite its longstanding clinical application, recent comparative studies consistently reveal its limitations in diagnostic precision, particularly when measured against newer cytokine-based or combinatorial markers.

CRP plays an important immunological role by acting as a pattern recognition molecule. It binds to phosphocholine residues on the surfaces of pathogens and apoptotic cells, thereby initiating the classical complement pathway and enhancing phagocytosis through interactions with Fcγ receptors on immune cells [51,52]. Through these mechanisms, CRP supports pathogen clearance and helps maintain tissue homeostasis [52,53].

#### 2.1.1. Differentiation of Infectious vs. Non-Infectious Neutropenic Fever

CRP has demonstrated good sensitivity for bacterial infections at fever onset, though its specificity remains modest. In one of the earliest pediatric FN studies, Santolaya et al. (1994) [23] showed that in febrile neutropenic children with cancer, a serum CRP concentration ≥40 mg/L obtained on Day 1 of fever identified culture-proven (Group I) bacterial infections with 100% sensitivity and 76.6% specificity. When ‘probable’ bacterial episodes (Group II) were analyzed together with proven cases (Groups I + II), sensitivity decreased modestly to 94.5%, whereas specificity remained virtually unchanged at 76.7%. On Day 2, the same 40 mg/L cutoff still detected culture-proven infections with 100% sensitivity, but specificity rose to 90% (Day 2 accuracy for the combined Groups I + II was not reported). El-Maghraby et al. (2007) [42] reported that a CRP cutoff of ≥90 mg/L yielded a sensitivity of 69.5% and a specificity of 73.1% for distinguishing febrile neutropenic episodes with any documented infection (clinical focus and/or bacteremia) from unexplained fever. Hence, while CRP showed moderate diagnostic accuracy, its sensitivity alone was insufficient to rule out non-infectious causes.

Kitanovski et al. (2006) [11] evaluated CRP, PCT, and IL-6 in febrile neutropenic children, demonstrating CRP’s inferior Day 1 AUC (0.649) compared to IL-6 (AUC = 0.867; *p* < 0.05) and PCT (AUC = 0.832; *p* > 0.05). While CRP improved by Day 3 (AUC = 0.802), PCT achieved the highest accuracy (AUC = 0.906). IL-6’s initial superiority (sensitivity: 87.5%, specificity: 86.0%) declined over time, whereas PCT’s high Negative Predictive Value (NPV) (97.3%) and dynamic performance reinforced its clinical utility. Similarly, Nahar et al. (2023) [8] reported that CRP showed moderate diagnostic accuracy (AUC = 0.697; 95% CI: 0.54–0.855), underperforming compared to PCT (AUC = 0.797; 95% CI: 0.651–0.943). PCT’s superiority was further supported by significantly higher median levels in bacteremic patients (26.10 μg/L vs. 0.78 μg/L; *p* = 0.002) and its >2 μg/L cutoff association with bacteremia. The study concluded PCT is a ‘superior early biomarker’ for bacterial detection in pediatric FN.

#### 2.1.2. Differentiation of Infectious Agent (GNB, GPB, Viral)

CRP demonstrates limited utility in identifying high-risk bacterial infections, particularly Gram-negative bacteremia, in immunocompromised pediatric populations. Xu et al. (2019) [27] evaluated biomarkers in 3118 febrile episodes among pediatric cancer patients and demonstrated CRP’s poor discriminative power for Gram-negative bacteremia (AUC = 0.56), significantly lower than IL-6 (AUC = 0.77) and IL-10 (AUC = 0.81). In multivariate analysis, CRP failed to independently predict GNB (*p* = 0.292) or septic shock (*p* = 0.094), whereas IL-6 (RR = 3.02–8.21) and IL-10 (RR = 4.28–5.77) were robust independent predictors (*p* < 0.001). The combination of IL-6 <185 pg/mL and IL-10 <20 pg/mL identified low-risk patients with a septic shock rate of only 0.7%, suggesting these cytokines enable early risk stratification in febrile pediatric cancer patients. Similarly, Gupta et al. (2020) [33] reported no significant difference in CRP between GNB (median: 60.7 mg/L) and GPB (85.5 mg/L; *p* = 0.796), consistent with its poor discriminatory power. While CRP was elevated in 81.3% of febrile neutropenia episodes (including sterile cultures), its levels did not reflect the microbial etiology. The study emphasized IL-6’s superior specificity for GNB (*p* = 0.017), underscoring CRP’s limited utility in guiding targeted therapy.

#### 2.1.3. Risk Stratification and Antimicrobial Policy

Although not sufficiently specific to guide etiologic diagnosis, CRP plays a smentary role in risk stratification and may aid in triaging patients for further diagnostic workup or escalation of antimicrobial therapy. Martinez-Albarran et al. (2009) [9] prospectively analyzed 54 febrile neutropenic children, showing CRP (cutoff: 9.06 mg/dL) had 77.7% sensitivity for high-risk infections but modest specificity (72.2%). PCT (cutoff: 0.67 ng/mL) was superior for confirmation (specificity: 80.5%; sensitivity: 72.2%). The study concluded CRP is better for screening, while PCT’s higher specificity makes it preferable for definitive diagnosis. Baraka et al. (2018) [41] demonstrated CRP’s moderate accuracy (AUC = 0.75 at ≥105 mg/L; sensitivity 77.8%, specificity 66.7%), significantly underperforming versus presepsin (AUC = 0.996 at ≥951 pg/mL) and PCT (AUC = 0.92 at ≥2.86 ng/mL) (*p* < 0.001). The study concluded presepsin’s near-perfect AUC (0.996) and specificity (100%) made it the optimal biomarker for bacteremia detection in febrile neutropenia.

Furthermore, Purkayastha et al. (2016) [40] reported that CRP, at a cutoff of ≥110 mg/L, demonstrated very poor sensitivity (13.3%) but relatively high specificity (77.2%) and NPV (77.2%) for bacteremia. In contrast, PCT, at a cutoff of ≥0.25 ng/mL, exhibited much higher sensitivity (73.3%) and NPV (83.3%), though with markedly lower specificity (29.4%). The authors concluded that PCT was a more sensitive marker for bacterial infection in febrile neutropenic children and recommended its routine use, despite low specificity, while CRP’s low sensitivity renders it suboptimal for early detection. Moreover, Araujo et al. (2017) [3] found no significant CRP differences between sepsis (estimated mean: 106.1 mg/L) and non-sepsis groups (52.9 mg/L; *p* = 0.14) in mixed models, unlike IL-6 (181.1 vs. 24.8 pg/mL; *p* < 0.001). Özdemir et al. (2019) [39] reported that serial CRP measurements (cutoffs: Day 1 = 2.5 mg/dL, Day 2 = 4 mg/dL, Day 7 = 7 mg/dL) effectively distinguished culture-positive from culture-negative febrile neutropenia episodes (*p* < 0.05 for all days), with AUCs ranging from 0.742 to 0.807. CRP levels increased progressively in bacteremic cases (Day 1 median: 6.79 mg/dL → Day 2: 12.0 mg/dL), supporting its utility in monitoring infection trends during treatment. Karakurt et al. (2014) [30] observed a higher median CRP in Fever of Unknown Origin (FUO) (5.41 mg/dL) than in microbiologically (4.93 mg/dL) or clinically proven infections (3.7 mg/dL; *p* = 0.06), contradicting conventional expectations. With only six microbiologically confirmed cases and 70% solid-tumor patients, the study underscores CRP’s inconsistent diagnostic utility in febrile neutropenia, particularly for infection confirmation.

Nevertheless, CRP retains clinical relevance when incorporated into multi-analyte models or serial monitoring protocols. When interpreted in the context of clinical signs, trends over time, and in conjunction with cytokine markers (e.g., IL-6, IL-8), CRP contributes meaningfully to patient stratification, particularly in institutions where access to molecular diagnostics is constrained.

### 2.2. Procalcitonin

Procalcitonin is a 116-amino acid precursor of the hormone calcitonin and is recognized as a reliable biomarker for bacterial infections. Under physiological conditions, PCT is secreted in minute quantities (<0.1 ng/mL) by thyroid C cells. However, in the presence of bacterial infections, systemic PCT production is markedly upregulated in non-neuroendocrine tissues such as hepatocytes, leukocytes, and pulmonary epithelium. This induction is stimulated by bacterial endotoxins—particularly, lipopolysaccharide (LPS)—and pro-inflammatory cytokines including IL-1β, TNF-α, and IL-6 [54].

PCT differs from CRP by its greater specificity for bacterial infections. While CRP may rise in response to various inflammatory stimuli, PCT levels remain low in viral infections due to suppression by interferon-gamma (IFN-γ). This distinction supports the clinical use of PCT in diagnosing bacterial sepsis, lower respiratory tract infections, and other systemic infections [55].

#### 2.2.1. Differentiation of Infectious vs. Non-Infectious Neutropenic Fever

PCT has consistently demonstrated superior performance over CRP in distinguishing infectious from non-infectious causes of fever in children with neutropenia. The study by Baraka et al. (2018) [41] demonstrated PCT’s strong diagnostic utility for bacteremia (AUC = 0.92 at ≥2.86 ng/mL; sensitivity = 100%, specificity = 81%), outperforming CRP (AUC = 0.75). Presepsin showed near-perfect accuracy (AUC = 0.996 at ≥951 pg/mL) for combined bacteremia/clinically proven infections, with significantly higher AUCs than PCT and CRP (*p* < 0.001). The study concluded presepsin was superior for bacterial infection detection, while PCT’s high sensitivity made it valuable for ruling out bacteremia. Hatzistilianou et al. (2010) [7] reported PCT’s superior diagnostic performance (94% sensitivity, 96.5% specificity; AUC = 0.875 at ≥2 ng/mL) in neutropenic bacterial infections, significantly outperforming CRP (AUC = 0.702) and IL-8 (AUC = 0.750; *p* < 0.01). A >30% PCT decrease within 48 h correlated with effective antibiotic therapy, while CRP and cytokines showed delayed responses. The study concluded PCT is optimal for early diagnosis and therapy monitoring.

These findings position PCT as a dynamic monitoring tool rather than a standalone diagnostic marker in this population. Serial PCT monitoring enhances its clinical utility. However, the temporal dynamics of PCT reveal limitations in longitudinal monitoring. Özdemir et al. (2019) [39] observed that PCT maintained higher median levels in culture-positive episodes (Day 1: 0.60 vs. 0.21 ng/mL; Day 2: 0.62 vs. 0.20 ng/mL) but exhibited declining diagnostic accuracy (AUC: 0.722 → 0.691). In contrast, CRP’s discriminatory performance improved (AUC: 0.758 → 0.807). The study concluded that neither biomarker is sufficient for standalone diagnosis, but CRP’s longitudinal trends may better reflect infection progression. The cohort strictly compared culture-confirmed bacteremia (*n* = 13) vs. culture-negative fever (*n* = 34), excluding ambiguous infection categories.

Furthermore, van der Galien et al. (2018) [28] reported PCT ≥ 0.25 ng/mL had 93.3% sensitivity (95% CI: 68.1–99.8%) but limited specificity (42.8%) for bacterial infections in pediatric febrile neutropenia. The median PCT was higher in bacteremic patients (1.00 vs. 0.32 ng/mL; *p* = 0.021). The study did not evaluate PCT kinetics beyond 24 h or correlate trends with disease severity, focusing instead on static diagnostic performance. The authors concluded PCT’s high sensitivity supports its role in infection screening but requires combination with other biomarkers for definitive diagnosis.

The study by Kitanovski et al. (2006) [11] demonstrated PCT’s robust early performance (Day 1 AUC = 0.832, sensitivity = 93.8%, NPV = 97.3% at >0.55 μg/L) for bacteremia/clinical sepsis, with accuracy improving to AUC = 0.906 by Day 3. IL-6 showed superior initial discrimination (Day 1 AUC = 0.867, specificity = 86.0%) but declined by Day 3 (AUC = 0.789). The study concluded that PCT’s rising AUC and exceptional NPV make it ideal for longitudinal monitoring, while IL-6’s early specificity may complement initial screening.

In pediatric leukemia cohorts, Nahar et al. (2023) [8] reported that PCT provided good diagnostic accuracy for bacteremia (AUC = 0.797; 95% CI: 0.651–0.943), significantly outperforming CRP (AUC = 0.697; 95% CI: 0.54–0.855). A PCT cutoff > 2 μg/L was significantly associated with bacteremia, with median PCT levels markedly higher in bacteremic cases (26.10 μg/L) compared to non-bacteremic cases (0.78 μg/L; *p* = 0.002). The findings indicated that PCT may serve as a superior early biomarker over CRP in predicting bacterial infection in children with febrile neutropenia and acute leukemia. Nevertheless, the relatively small bacteremia cohort (*n*= 12 of 58 patients) limited definitive conclusions about the standalone utility of PCT.

Srinivasan et al. (2021) [13] demonstrated PCT’s diagnostic primacy, with an AUC of 0.745—significantly outperforming interleukin-6 (IL-6; AUC = 0.574) and interleukin-8 (IL-8; AUC = 0.551). At a cutoff >2 ng/mL, PCT achieved 91% specificity and 88% NPV, though its moderate sensitivity (63%) highlighted challenges in detecting all infections. While IL-6 and IL-8 showed utility as exclusion tools (NPVs: 73–80%), their poor specificity (≤57%) and low sensitivity (≤54%) limited their standalone use. This reinforced PCT’s role as a superior confirmatory marker for bacterial etiology, albeit with a need for complementary tests to address its sensitivity gap.

The high NPV of PCT was further corroborated by Zareifar et al. (2020) [38], who reported an NPV of 91% at a cutoff ≥ 0.70 ng/mL. However, the study also noted a low PPV (47.5%), emphasizing PCT’s reliability for ruling out infections rather than confirming them. With an AUC of 0.74, PCT’s moderate accuracy underscored the necessity of integrating clinical context and additional biomarkers to improve diagnostic certainty.

These findings align with earlier work by Purkayastha et al. (2016) [40], which revealed PCT’s critical trade-offs at lower cutoffs (≥0.25 ng/mL). Despite an acceptable NPV (83.3%), the abysmal specificity (29.4%) and high false-positive rate (47/67 culture-negative cases) highlighted the risks of overreliance on PCT alone. The study’s low Positive Predictive Value (PPV) (18.6%) and non-significant odds ratio (1.15, *p* > 0.05) further underscored the biomarker’s limited predictive power in small bacteremic subgroups. Notably, PCT’s sensitivity (73.3%) surpassed CRP’s (13.3%), but its clinical utility remained contingent on interpretation alongside clinical findings, particularly in non-bacteremic fevers.

#### 2.2.2. Differentiation of Infectious Agent (GNB, GPB, Viral)

PCT demonstrates suboptimal performance in identifying Gram-negative bacteremia in immunocompromised pediatric populations. Xu et al. (2019) [27] evaluated biomarkers in 3118 febrile episodes among pediatric cancer patients, showing PCT’s limited discriminatory capacity for GNB (AUC = 0.68), significantly inferior to IL-10 (AUC = 0.81, *p* < 0.001) and IL-6 (AUC = 0.77, *p* < 0.001). Multivariate analysis confirmed PCT’s non-significant predictive value for GNB (RR = 1.32, *p* = 0.105), while IL-10 (RR = 5.77) and IL-6 (RR = 3.02) were robust independent predictors (*p* < 0.001). Although PCT showed stronger association with septic shock (RR = 2.24, *p* = 0.001), IL-6 (RR = 8.21) and IL-10 (RR = 4.28) demonstrated superior predictive capacity for this critical outcome, emphasizing the clinical utility of cytokine-based risk stratification in febrile pediatric cancer patients. This aligns with findings by Kitanovski et al. (2006) [11], who reported PCT’s Day 1 AUC of 0.832 (cutoff > 0.55 μg/L) for bacteremia detection, with a sensitivity of 93.8% and NPV of 97.3%. While PCT levels were 3–5× higher in Gram-negative cases, the difference from Gram-positive bacteremia was non-significant (*p* > 0.05), attributed to the small cohort (n = 15 bacteremia) and high proportion of virulent streptococcal infections (60% of GPB). The study highlighted PCT’s rising accuracy by Day 3 (AUC = 0.906) but concluded it lacks proven discriminatory power for pathogen subtypes in small cohorts. Notably, IL-6 outperformed PCT on Day 1 (AUC = 0.867) but declined by Day 3.

#### 2.2.3. Risk Stratification and Antimicrobial Policy

Procalcitonin demonstrates clinical utility in risk stratification and antimicrobial stewardship for febrile neutropenic patients. Nonkulovski et al. (2020) [37] reported PCT ≥ 2 ng/mL at admission achieved 100% sensitivity for sepsis in 20 pediatric cancer patients, with PCT > 10 ng/mL identifying the sole septic shock case (5% mortality). A ≥50% PCT reduction by Days 3–5 correlated with therapeutic response, enabling antibiotic de-escalation in 75% (15/20) by Day 14. Persistently elevated PCT (>10 ng/mL) predicted fatal outcomes (1/20). All patients received protocolized dual antibiotics (Meropenem + Amikacin/Vancomycin), with PCT outperforming CRP in early detection. Further supporting its diagnostic utility, Martinez-Albarran et al. (2009) [9] demonstrated PCT’s superior specificity (80.5% at 0.67 ng/mL) over CRP (72.2% at 9.06 mg/dL) for bacterial infection confirmation in 54 pediatric febrile neutropenia episodes, with both markers significantly differentiating high- and low-risk groups (PCT: *p* = 0.003; CRP: *p* = 0.001). While CRP’s higher sensitivity (77.7%) supports screening, the study did not assess PCT’s role in antibiotic adjustments. Notably, all deaths (4/18 high-risk) occurred in septic shock patients with elevated PCT/CRP, underscoring their potential prognostic value, despite the absence of formal statistical correlation with mortality.

Recent studies have progressively refined the use of biomarker combinations for infection risk stratification in febrile neutropenia. Doerflinger et al. (2021) [16] prospectively validated a dual-marker model combining PCT (≥0.425 ng/mL) and IL-10 (≥4.37 pg/mL), which achieved 100% sensitivity and 89% specificity for bacteremia prediction. This model retained robust performance on cross-validation (87.5% sensitivity, 87.3% specificity) and enabled reclassification of 80% of episodes (63/79) as low-risk without missing any bacteremia. Notably, PCT exhibited an 11-fold rise by Day 2 in confirmed cases, underscoring its kinetic utility. However, the authors cautioned that the small bacteremia subgroup (n = 8) necessitated further prospective validation before clinical adoption.

This builds on earlier work by Miedema et al. (2010) [20], who tested a similar approach with PCT (>0.25 ng/mL) and IL-8 (>60 ng/L). While this combination also achieved 100% sensitivity, its lower specificity (52%) highlighted challenges in distinguishing bacterial infections from confounders like mucositis (which elevated IL-8; *p* < 0.01). Moreover, PCT’s delayed rise (significant only at 24–48 h; *p* = 0.047) underscored the importance of serial measurements. Critically, Miedema et al. (2010) [20] emphasized that while their IL-8/PCT combination showed promise for risk stratification in febrile neutropenia, the model’s clinical utility was limited by IL-8’s lack of specificity for bacterial infection, particularly given its elevation in mucositis, and stressed the need for further validation studies.

### 2.3. Interleukin-6

Interleukin-6 is a pleiotropic cytokine that plays a central role in immune regulation, inflammation, and hematopoiesis. It is secreted by macrophages, dendritic cells, and T lymphocytes in response to pathogen-associated molecular patterns and tissue damage. IL-6 exerts its biological effects through two distinct signaling pathways: classical signaling, which involves membrane-bound IL-6 receptors (mIL-6Rs) predominantly expressed on immune cells, and trans-signaling, mediated by soluble IL-6 receptors (sIL-6Rs), which allows IL-6 to act on a broader range of non-immune cells, including endothelial and epithelial cells. This dual-signaling mechanism enables IL-6 to coordinate immune responses across multiple tissues, positioning it as a key mediator of host defense and inflammatory homeostasis [56]. In bacterial infections, IL-6 is among the earliest cytokines released, initiating the acute-phase response. It stimulates hepatocytes to produce CRP and serum amyloid A (SAA), key acute-phase reactants involved in opsonization and microbial clearance [57]. Additionally, IL-6 promotes neutrophil recruitment and activation—essential processes for containing and eradicating bacterial pathogens [58]. Numerous studies have demonstrated a strong association between elevated IL-6 levels and adverse outcomes in bacterial sepsis, including multi-organ dysfunction and mortality [59,60,61].

#### 2.3.1. Differentiation of Infectious vs. Non-Infectious Neutropenic Fever

Multiple studies have demonstrated IL-6’s high sensitivity and specificity in detecting bacterial infections at the onset of fever. Riikonen et al. (1992) [19] found IL-6 detectable in the majority of febrile neutropenia episodes but elevated in only 15%, with no significant differences between bacteremia and other causes (*p* > 0.05). IL-6 showed no discriminative power for bacteremia and correlated weakly with CRP only on Days 1–2 (*r* = 0.25, *p* = 0.02). While SAA achieved 100% sensitivity for bacteremia (vs. CRP’s 68%), neither marker reliably predicted individual risk. The study emphasized that cytokines (IL-6, TNF-α, IL-1β) reflected fever severity rather than bacterial etiology, and SAA—though superior to CRP—required further validation for clinical use. Abrahamsson et al. (1997) [12] reported IL-6 > 50 pg/mL had 74% sensitivity for bacteremia but only 49% specificity, with a 45% PPV. While more sensitive than CRP (29%), IL-6’s elevation in 57% of FUO episodes (37/65) and failure to detect 26% of bacteremia (9/34) precluded standalone use for antibiotic decisions. IL-6 correlated weakly with CRP on Day 0 (*r* = 0.26) but strongly by Day 2 (*r* = 0.53), reflecting its earlier rise. The study emphasized IL-6’s potential for low-risk stratification but cautioned against withholding antibiotics in neutropenia without validation. TNF-α levels were suppressed in neutropenic patients (*p* < 0.001), suggesting chemotherapy blunts this response. These findings were significantly expanded by Kitanovski et al. (2006) [11], who demonstrated that IL-6 > 235.1 pg/mL on Day 1 had superior diagnostic accuracy for bacteremia/clinical sepsis (AUC = 0.867, sensitivity 87.5%, specificity 86.0%, NPV 95.6%) compared to CRP (AUC = 0.649, *p* < 0.05), though its utility diminished by Day 3 (AUC = 0.789). PCT maintained high accuracy throughout (AUC = 0.832–0.906), with sequential measurements improving its predictive value. Notably, PCT levels were 3–5× higher in Gram-negative bacteremia (NS), while IL-6’s early peak and subsequent decline reflected its role as an acute-phase marker. The study emphasized PCT’s reliability for serial monitoring but cautioned about small sample size (16 bacteremia) and IL-6’s detection limits (max 300 pg/mL). Tapia et al. (2021) [6] developed a four-marker panel (IL-6, G-CSF, Flt-3L, IP-10) achieving AUC = 0.839 (75% sensitivity, 81% specificity) for distinguishing infectious (bacterial/viral) from non-infectious HRFN at day 4. IL-6 was significantly higher in infections (median 80.81 vs. 13.37 pg/mL; *p* = 0.002), while G-CSF showed 14.6-fold elevation in Febrile Neutropenia with Detected Etiological Agent (FN-DEA) (*p* < 0.001). IP-10 had viral-specific utility (AUC = 0.702). Fungal cases (n = 8) were excluded from validation due to a limited sample size. The study emphasized the panel’s research (not clinical) applicability pending prospective trials.

Xia et al. (2016) [35] demonstrated that IL-6 was significantly elevated in culture-positive sepsis cases compared to controls (median 688.2 pg/mL vs. 6.8 pg/mL; *p* < 0.001). Using a cutoff of >16.6 pg/mL, IL-6 achieved 92.8% sensitivity, outperforming CRP (73.1%) and procalcitonin (PCT; 33.8%) in bacteremia detection (*p* < 0.01). For septic shock, IL-6 yielded the highest area under the ROC curve (AUC = 0.875), compared to CRP (0.648) and PCT (0.726). IL-10 also showed elevated diagnostic utility with 82.2% positivity in sepsis cases, though its comparative statistics were less emphasized. Importantly, results were obtainable within five hours via flow cytometry, allowing earlier intervention than blood cultures. While the study supports IL-6’s strong diagnostic performance, the authors note limitations including its retrospective, single-center design, and advocate for further prospective validation.

#### 2.3.2. Differentiation of Infectious Agent (GNB, GPB, Viral)

IL-6 has demonstrated meaningful discriminatory power in differentiating Gram-negative from Gram-positive bacterial infections.

Gupta et al. (2020) [33] observed higher IL-6 levels in Gram-negative bacteremia (median 169 pg/mL, IQR: 124–2600) compared to sterile episodes (median 52 pg/mL; *p* = 0.017), while Gram-positive cases (*n* = 2) showed lower IL-6 (median 17.5 pg/mL). CRP lacked discriminative power (Gram-negative: 60.7 mg/L; Gram-positive: 85.5 mg/L; sterile: 44.2 mg/L; *p* = 0.796). All Microbiologically Proven Infection (MDI) cases had profound neutropenia (ANC < 100/μL), and IL-6 >100 pg/mL predicted mortality (4/4 fatal cases). These findings were corroborated by Xu et al. (2019) [27] in the largest prospective cohort to date (3118 febrile episodes in pediatric cancer patients). They identified IL-6 ≥ 185 pg/mL as an independent predictor of Gram-negative bacteremia (AUC = 0.77, RR = 3.02, *p* < 0.001), significantly outperforming CRP (AUC = 0.56). IL-10 demonstrated even superior individual performance for GNB detection (AUC = 0.81, RR = 5.77, *p* < 0.001). Notably, the combination of IL-6 < 185 pg/mL and IL-10 < 20 pg/mL identified low-risk patients with a septic shock rate of only 0.7%, suggesting clinical utility for risk stratification in febrile pediatric cancer patients. Soker et al. (2001) [18] observed significantly higher median IL-6 levels in Gram-negative bacteremia (166 pg/mL; range: 21–1780) compared to Gram-positive cases (26 pg/mL; range: 16–33; *p* = 0.042) in a small pediatric cohort (*n* = 11 bacteremia). IL-8 (*p* = 0.023) and soluble IL-2 receptor (*p* = 0.006) also showed discriminatory potential. While these findings suggested that early cytokine profiles might support timely initiation of empirical antibiotics, the overlapping IL-6 values (e.g., up to 1780 pg/mL in GNB vs. 33 pg/mL in GPB) and the absence of interventional outcome data limited their use for pathogen-specific therapy. The study emphasized IL-6’s association with Gram-negative infections but noted that TNF-α and IL-1β lacked discriminatory power (*p* > 0.05). Three deaths occurred (two in Gram-negative infections), highlighting the need for larger-scale validation.

#### 2.3.3. Risk Stratification and Antimicrobial Policy

IL-6 is one of the most extensively explored cytokines for risk stratification in pediatric FN. Diepold et al. (2008) [14] identified IL-6 > 42 pg/mL at fever onset as predictive of high-risk episodes (sepsis or fever ≥5 days) with 90% sensitivity and 85% specificity. Sepsis cases (median IL-6 = 326 pg/mL) differed significantly from low-risk episodes (median 21 pg/mL; *p* < 0.01). IL-6 outperformed CRP (specificity 59%), with PPV 94% and NPV 77%. IL-8 (cutoff 30 pg/mL) showed lower specificity (59%). The study proposed risk stratification but noted limitations (small sepsis subgroup, single-center data). Kharya et al. (2007) [10] observed that IL-6 > 20 pg/mL identified all sepsis cases (15/15) and deaths (6/6) in febrile neutropenia, with 42.5% specificity (20/47 non-sepsis cases below cutoff). PCT > 1 ng/mL missed three infections (one aspergillosis, one Candida, one abscess). While combining IL-6 < 20 pg/mL with PCT < 1 ng/mL was proposed for low-risk stratification, this approach lacked validation. The study also noted IL-6’s superior AUC at 48 h (0.77 vs. 0.73 at 0 h) and TNF-α/hsCRP’s non-significance (*p* > 0.05). Araujo et al. (2017) [3] demonstrated IL-6 > 170 pg/mL at fever onset discriminated sepsis in neutropenic children with 69% sensitivity and 95% specificity (AUC = 0.87). Combining IL-6 > 50 pg/mL with PCT > 100 pg/mL increased sensitivity to 85% while maintaining 82% specificity. Filgrastim recipients had a higher median IL-6 (132 vs. 39.5 pg/mL; *p* = 0.007), yet IL-6 retained diagnostic value (AUC = 0.78 with filgrastim vs. 0.9 without). IL-8 showed superior performance (AUC = 0.86) and was unaffected by filgrastim. The study highlighted IL-6/PCT’s additive value but noted limitations (small sample, single-center design). Van der Galiën et al. (2018) [28] identified IL-6 < 60 pg/mL + PCT < 0.25 ng/mL as a potential low-risk criterion (NPV 100%, identifying 41% of episodes at T0). One perianal abscess case with PCT < 0.25 ng/mL underscored the need for clinical correlation. The study proposed combining these biomarkers with clinical factors in future validation, noting IL-8’s superior performance (AUC 0.86) and strong correlation with IL-6 (*r* = 0.78–0.96). Limitations included the small bacterial infection subgroup (*n* = 18) and the lack of interventional data.

Beyond binary risk classification, IL-6 has also been correlated with fever duration and treatment response. Karakurt et al. (2014) [30] observed higher median IL-6 (142 vs. 82 pg/mL; *p* = 0.02) in febrile neutropenia episodes with fever >3 days. IL-8 (*p* = 0.01) and sTNFRII (*p* = 0.04) showed similar predictive value. IL-6 did not correlate with severe infection risk (*p* = 0.11 for microbiologically vs. clinically proven infections) or treatment outcomes in multivariate analysis. PCT > 1 ng/mL predicted longer fever duration (5.8 vs. 2.6 days; *p* = 0.014). Limitations included the small sample size (*n* = 6 bacteremia) and no ICU/mortality assessment

In contrast to these findings, other authors did not confirm the potential of IL-6 as an independent prognostic marker in pediatric febrile neutropenia. Srinivasan et al. (2021) [13] demonstrated that IL-6 levels did not significantly differ between episodes with (82.45 pg/mL) and without documented infection (76.03 pg/mL; *p* = 0.81). While a threshold of 50 pg/mL provided a modest NPV of 80%, its sensitivity (54%) and specificity (57%) were clinically inadequate. In contrast, PCT at <2 ng/mL showed superior performance (NPV 88%, specificity 91%). The authors recommended using these biomarkers collectively to identify low-risk patients for early discharge, given their favorable NPVs (IL-6: 80%, PCT: 88%, IL-8 < 130 pg/mL: 73%)—though this strategy required validation beyond their single-center study with limited documented infections (*n* = 11).

These findings align with Aggarwal et al. (2013) [15], who reported no significant difference in mean IL-6 levels between high-risk (95.2 pg/mL) and low-risk episodes (46.9 pg/mL; *p* = 0.11). While IL-6 < 10 pg/mL achieved an NPV of 82% for excluding severe infection, higher thresholds (>100 pg/mL) performed poorly (sensitivity 36%, specificity 24%, PPV 11%). The study emphasized IL-8’s superior NPV (88% at <15 pg/mL) and suggested that combining IL-6, IL-8, and TNF-α could support antimicrobial stewardship decisions, albeit with similar limitations (*n* = 17 high-risk episodes).

The transient nature of IL-6’s diagnostic signal was further elucidated by Doerflinger et al. (2021) [16], who found no significant difference in IL-6 levels between bacteremic and non-bacteremic episodes (AUC = 0.625; *p* = 0.249). Despite high specificity (90%) at >356 pg/mL, sensitivity remained low (50%), and levels declined 6.1-fold by Day 2, undermining its dynamic utility. The study highlighted the superiority of PCT (AUC = 0.842) and IL-10 (AUC = 0.826), with their combination achieving 100% sensitivity and 89% specificity—identifying 80% of episodes (*n* = 79) as low risk without missing bacteremia. MIP-1β (AUC = 0.780) also outperformed IL-6, reinforcing the need for multimarker strategies.

Multimarker strategies enhance IL-6’s prognostic utility. In the largest pediatric cohort to date, Xu et al. (2019) [27] identified IL-6 ≥ 185 pg/mL as superior for septic shock prediction (AUC = 0.89, 84.4% sensitivity, 79.3% specificity) versus PCT (AUC = 0.78) and CRP (AUC = 0.65) in 3118 febrile episodes. The threshold detected 87% of septic shock cases in bloodstream infections. IL-10 (AUC = 0.87) showed comparable performance, and their combination reduced septic shock risk to 0.7% when both were below cutoffs. The study highlighted IL-6/IL-10’s predictive value for Gram-negative bacteremia (AUCs = 0.77–0.81) but noted limitations (single-center design, static measurements).

IL-6 is a contextually useful biomarker in pediatric oncological patients with febrile illness, with demonstrated value in early infection detection (e.g., Tapia et al. (2021) [6]: AUC = 0.743) and risk stratification. The combination of IL-6 < 185 pg/mL with IL-10 < 20 pg/mL effectively identified low-risk patients (septic shock rate 0.7%), enabling early risk stratification. Its standalone utility is limited by rapid normalization (Doerflinger et al. (2021) [16]: 6.1-fold decline by Day 2). IL-6 demonstrated superior discriminatory power over CRP for Gram-negative bacteremia detection (Gupta et al. (2020) [33]: 169 vs. 17.5 pg/mL), with IL-6 > 100 pg/mL predicting mortality in septic shock. However, study limitations included the small sample size and inability to perform serial IL-6 measurements due to financial constraints, highlighting the need for larger studies integrating multiple biomarkers and cost-effectiveness analysis. Clinical implementation requires validation in diverse cohorts.

### 2.4. Interleukin-8

Interleukin-8, a member of the chemokine family, plays a critical role in orchestrating the innate immune response, particularly by recruiting and activating neutrophils during acute bacterial infections. It is secreted by multiple cell types, including macrophages, neutrophils, and endothelial cells, and exerts its effects primarily through CXCR1 and CXCR2 receptors, triggering intracellular signaling cascades such as PI3K and PLC pathways that mediate chemotaxis, cell survival, and phagocytosis [62,63].

Elevated IL-8 levels are characteristic of bacterial infections and correlate with disease severity, especially in sepsis [64].

#### 2.4.1. Differentiation of Infectious vs. Non-Infectious Neutropenic Fever

IL-8 demonstrates rapid elevation at the onset of fever, often preceding CRP and coinciding with PCT in time course. However, the diagnostic utility of IL-8 in differentiating infectious from non-infectious febrile neutropenia remains contentious. Srinivasan et al. (2021) [13] found no significant difference in IL-8 levels between pediatric FN episodes with microbiologically confirmed infections (median: 613.6 pg/mL) and non-infectious fever (263.7 pg/mL; *p* = 0.41), with an AUC of 0.551—barely exceeding random chance. At a cutoff of 130 pg/mL, IL-8 exhibited poor sensitivity (45%) and specificity (49%), reinforcing its inadequacy as a standalone marker. However, its NPV of 73% suggested potential for ruling out severe infections when combined with clinical assessment. Aggarwal et al. (2013) [15] prospectively evaluated IL-8 in 52 FN episodes among pediatric hematological malignancy patients, stratifying cohorts into low-risk (no infection focus) and high-risk (clinical/microbiological infection) groups. Despite observing wider IL-8 variability in high-risk cases (mean 544 vs. 219 pg/mL in low-risk), the difference lacked statistical significance (*p* = 0.44). At a 300 pg/mL cutoff, IL-8 showed marginal predictive value (41% high-risk vs. 17% low-risk, *p* = 0.06), with poor sensitivity (46%) and specificity (26%). However, its NPV of 88% at 15 pg/mL underscored the potential for excluding severe infections, mirroring Srinivasan’s conclusions about the potential for ruling out severe infections. Doerflinger et al. (2021) [16] confirmed this narrative in a prospective analysis of 79 pediatric FN episodes, finding no significant difference in IL-8 levels between bacteremia (optimal ROC-derived cutoff >665.5 pg/mL) and non-bacteremia cases (AUC 0.666, *p* = 0.127). IL-8 demonstrated poor sensitivity (50%), despite moderate specificity (80%), with no improvement when combined with clinical variables like platelet count or fever severity. These findings are consistent with Srinivasan and Aggarwal further questioning its standalone utility. Jaing et al. (2020) [24] prospectively evaluated 30 pediatric Hematopoietic Stem Cell Transplantation (HSCT) recipients with febrile neutropenia, observing comparable IL-8 levels between the infectious (mean: 95.4 ng/L) and non-infectious groups (mean: 94.9 ng/L; *p* = 0.99), even when applying a 60 ng/L cutoff. IL-8 demonstrated moderate specificity (77.3%) for infectious complications; however, its diagnostic sensitivity could not be meaningfully assessed, as no bacteremia cases occurred in the cohort. IL-8 levels decreased within 72 h considerably earlier than CRP in the absence of documented bacteremia, potentially informing antibiotic de-escalation, though its moderate specificity (77.3%) and lack of bacteremia cases in this cohort preclude standalone diagnostic use. A statistically significant correlation between IL-8 and CRP levels was observed (r = 0.289, *p* = 0.039). The study also emphasized IL-8’s interpretive limitations in the post-transplant setting, particularly where tumor-related and mucositis-driven inflammatory responses may confound biomarker profiles. These findings reinforce IL-8’s limited standalone diagnostic value but support its contextual use within antimicrobial stewardship strategies.

Contrasting these findings, Miedema et al. (2010) [20] reported that IL-8 concentrations ≥60 ng/L achieved 92% sensitivity for identifying bacterial infections in febrile neutropenic episodes, with median IL-8 levels significantly higher in infected patients (318.5 ng/L) compared to non-infected controls (55 ng/L; *p* = 0.002). The study further demonstrated that combining IL-8 with procalcitonin (PCT > 0.25 ng/mL) improved diagnostic performance to 100% sensitivity, albeit at the expense of specificity (52%). However, IL-8’s utility was confounded by non-infectious mucositis (*p* < 0.01), a limitation explicitly noted in the original discussion. El-Maghraby et al. (2007) [42] corroborated these findings in a cohort of pediatric FN patients, identifying IL-8 ≥ 62 pg/mL as a threshold for documented infections (microbiological or clinical) with 71% sensitivity (42/59) and 77% specificity (20/26). Median IL-8 concentrations were 125 pg/mL (range: 30–3120) in the infected cohort (20 microbiologically confirmed, 39 clinically documented) versus < 62 pg/mL in unexplained fever (*p* < 0.001). Although the standalone predictive values were moderate (PPV = 88%, NPV = 54%), diagnostic accuracy improved substantially when IL-8 was interpreted with CRP and MCP-1α: ≥2 elevated biomarkers (CRP ≥ 90 mg/L, IL-8 ≥ 62 pg/mL, MCP-1 ≥ 350 pg/mL) were observed in 78% of infected patients (46/59) versus 16% of non-infected cases (4/25). Akcay et al. (2021) [31] identified IL-8 ≥ 200 pg/mL as the optimal cutoff for bacteremia/sepsis (80% sensitivity, 65% specificity), with a high NPV (92%) supporting its rule-out utility. IL-8 was the only marker significantly elevated in B/S (*p* = 0.021), outperforming PCT, CRP, and IL-6 in this pediatric cohort.

Hatzistilianou et al. (2010) [7] further validated IL-8’s role in pediatric ALL patients with chemotherapy-induced neutropenia, demonstrating significantly higher IL-8 levels in bacterial infections (neutropenic: 145.4 pg/mL; non-neutropenic: 176.1 pg/mL) compared to viral/FUO episodes (58.2–42.5 pg/mL; *p* < 0.001). IL-8 was undetectable in afebrile controls (28 pg/mL; *p* < 0.001 vs. febrile groups). Despite IL-8’s moderate diagnostic accuracy (AUC = 0.750), it was outperformed by PCT (AUC = 0.875), underscoring its smentary role. Notably, IL-8 levels in neutropenic bacterial infections were lower than in non-neutropenic cases, suggesting neutrophil-independent production (e.g., endothelial cells). The study emphasized that IL-8’s dynamic profile—peaking on Day 2 (159–199 pg/mL), followed by gradual decline—aligns with fever resolution, though it lacks standalone specificity for bacterial etiology compared to PCT’s 94% sensitivity/96.5% specificity at 2 ng/mL.

#### 2.4.2. Differentiation of Infectious Agent (GNB, GPB, Viral)

Several studies have examined IL-8’s capacity to discriminate between Gram-negative and Gram-positive bacterial infections. Urbonas et al. (2011) [25] observed IL-8 concentrations 3.9–4.3 times higher in Gram-negative bloodstream infections compared to Gram-positive cases (*n* = 16 children), though no *p*-value was reported. The authors proposed IL-8 as a potential adjunct diagnostic tool for bacteremia in pediatric febrile neutropenia, pending further validation. Şahbudak Bal et al. (2017) [32] reported that IL-8 ≥ 61.3 pg/mL achieved 87.5% sensitivity, 74.5% specificity, and 97.4% NPV for identifying Gram-negative bacteremia/fungemia (*n* = 9 episodes, including 8 Gram-negative bacteremia and 1 Candida parapsilosis fungemia). IL-8 had the highest AUC (0.772, *p* = 0.008) for this subgroup, supporting its role in ruling out Gram-negative infections. Similarly, Soker et al. (2001) [18] reported median IL-8 levels of 916 pg/mL (126–4838) in Gram-negative bacteremia (*n* = 7) vs. 133 pg/mL (16–242) in Gram-positive cases (*n* = 4), a statistically significant difference (*p* = 0.023). The analysis excluded two fungemia episodes, and the authors proposed IL-8 as a smentary marker for differentiating bacterial etiologies during febrile neutropenia, alongside IL-6 and sIL-2R. Tapia et al. (2021) [6] reported that IL-8, though elevated in bacterial infections (median 130.52 vs. 38.69 pg/mL in FN-UO, *p* < 0.001), was not a key discriminator. Instead, IP-10 showed higher specificity for viral etiology (AUC = 0.702), while G-CSF (AUC = 0.665) better identified bacterial infections. The study did not evaluate IL-8’s specificity for Gram-negative pathogens.

#### 2.4.3. Risk Stratification and Antimicrobial Policy

IL-8’s rapid induction and high sensitivity make it valuable for early risk stratification in febrile neutropenic children. Nijhuis et al. (2005) [34] classified 18% of FN episodes (*n* = 36/196) as low risk using a model combining clinical parameters and IL-8 ≤ 60 ng/L (adjusted from 40 ng/L after safety validation). No bacteremia occurred in this group (100% NPV), enabling antibiotic withholding and shorter hospitalization (median 3 vs. 7 days; *p* < 0.0001). Dynamic IL-8 monitoring reclassified 14% of initially low-risk patients due to rising levels. Cost et al. (2013) [29] demonstrated that IL-8 < 1260 pg/mL combined with hemodynamic stability identified low-risk bacteremia cases with 90% sensitivity and 98% NPV, though two stable bacteremia cases were misclassified. IL-8 was the strongest multivariate predictor (OR = 6.06; 95% CI: 2.557–14.35) and outperformed existing clinical models (AUC = 0.81 vs. 0.61–0.69). The authors cautioned against standalone use due to false-negatives.

IL-8 also correlates with infection severity and treatment outcomes. Karakurt et al. (2014) [30] reported significantly higher median IL-8 levels in patients with fever > 3 days (472.11 vs. 97.53 pg/mL; *p* = 0.01) and those requiring treatment escalation (464.53 vs. 254.28 pg/mL; *p* = 0.048). While IL-8, IL-6, and sTNFRII predicted prolonged fever, no diagnostic cutoff was established. The study emphasized cytokines’ smentary role in monitoring febrile neutropenia, particularly in non-leukemic pediatric cancer patients. Diepold et al. (2008) [14] demonstrated that IL-6 (>42 pg/mL) was the strongest predictor of severe infection (90% sensitivity, 85% specificity, 94% PPV), while IL-8 (>30 pg/mL) exhibited lower specificity (59%) and sensitivity (87%). The authors concluded that IL-6 was the best predictor for identifying patients at risk of bacteremia or prolonged fever episodes, outperforming both IL-8 and CRP in diagnostic accuracy. Akcay et al. (2021) [31] reported IL-8’s specificity for bacteremia/sepsis was limited (65% at 200 pg/mL) due to elevations in non-B/S episodes (e.g., CMDI, FUO). While IL-8 demonstrated higher sensitivity (80%) than PCT (40%) and equal sensitivity to IL-6 (80%), its standalone PPV remained low (36%). The authors advocated combining IL-8 with PCT (specificity 88–100%) to improve risk stratification, noting IL-8’s high NPV (92%) for ruling out B/S. Araujo et al. (2017) [3] reported that IL-8 > 240 pg/mL combined with a high-risk clinical assessment achieved 100% specificity and PPV for sepsis, albeit with 69% sensitivity. The diagnostic accuracy improved to 85% sensitivity and 91% specificity (NPV 91%) when using a lower IL-8 cutoff (>100 pg/mL) combined with PCT > 100 pg/mL, underscoring the potential of biomarker synergy in infection stratification.

In conclusion, IL-8 may serve as a valuable biomarker for early detection and risk stratification in pediatric febrile neutropenia, particularly due to its rapid elevation profile and consistently high negative predictive value across multiple studies. Although its sensitivity varies widely depending on clinical context and study design, IL-8 has shown promise as an exclusionary tool and candidate for early discharge protocols. When used in combination with other biomarkers—particularly PCT or IL-10—its diagnostic accuracy and clinical utility are significantly enhanced. Nevertheless, variability in specificity and susceptibility to elevations in non-infectious conditions underscore the need for cautious interpretation and further standardization before routine clinical application.

### 2.5. Interleukin-10

Interleukin-10 (IL-10) is a key anti-inflammatory cytokine that regulates immune responses to prevent excessive tissue damage while maintaining control over infection. Secreted by various immune cells—including macrophages, T cells, and B cells—IL-10 suppresses pro-inflammatory cytokine production, primarily by inhibiting NF-κB activity. This immunomodulatory effect is mediated through IL-10 receptor (IL-10R) binding and downstream activation of the JAK-STAT pathway, specifically, STAT3, resulting in downregulation of inflammatory mediators such as TNF-α and IL-1β [65,66].

#### 2.5.1. Differentiation of Infectious vs. Non-Infectious Neutropenic Fever

IL-10 consistently demonstrates high diagnostic performance for bacterial infection and sepsis detection in febrile neutropenic patients. Doerflinger et al. (2021) [16] analyzed 79 FN episodes, showing PCT ≥ 0.425 ng/mL + IL-10 ≥ 4.37 pg/mL achieved 100% sensitivity (95% CI: 68.8–100%) and 89% specificity (95% CI: 80.0–95.0%) for bacteremia, correctly classifying all eight cases. The model outperformed clinical variables, and IL-10 levels declined 5-fold by Day 2 in bacteremia, suggesting therapeutic monitoring utility. The combination increased low-risk classification to 80% when added to clinical rules. Urbonas et al. (2012) [17] analyzed 36 FN episodes, showing IL-10 ≥ 18 pg/mL achieved 73% sensitivity (95% CI: 39–94%) and 92% specificity (95% CI: 74–99%) for bacterial sepsis (AUC = 0.87), with NPV = 83% and PPV = 86%. The study highlighted IL-10’s utility in excluding non-bacterial fever. Şahbudak Bal et al. (2017) [32] found IL-10 ≥ 5.04 pg/mL detected culture-confirmed bacteremia/fungemia (eight Gram-negative, five Gram-positive, one fungal; *n* = 14/59 episodes) with 92.9% sensitivity and NPV of 95.2% (AUC = 0.725, *p* = 0.003), though specificity was limited (44.4%). These findings underscore IL-10’s utility in ruling out confirmed infections, independent of pathogen type, despite its low PPV (34.2%). Cost et al. (2013) [29] found IL-10 levels were approximately 50-fold higher in bacteremic patients (846.3 vs. 16.9 pg/mL; *p* = 0.005), highlighting its association with bloodstream infections in univariate analysis. However, IL-10 was not retained in the final multivariate logistic regression model, which was dominated by IL-8 (OR = 6.06; 95% CI: 2.557–14.35). IL-8 achieved an AUC of 0.81, with 90% sensitivity and 98% negative predictive value, emerging as the most robust standalone predictor. While IL-10 demonstrated significance individually, its predictive contribution did not surpass IL-8, suggesting a more supportive role within broader biomarker panels for risk stratification. Kim et al. (2022) [26] observed IL-10 peaked at fever onset (Day 1: 27.4 pg/mL) but declined sharply by Day 4 (4.2 pg/mL; *p* = 0.001), mirroring IL-6 kinetics. Neither cytokine predicted prolonged fever (≥3 days; *p* = 1.000) or clinical outcomes in 10 bacteremia patients (3/10 with fever ≥ 3 days). The study framed IL-10 as an early-response marker, though limited by the small sample size (*n* = 10) and lack of non-infectious controls

#### 2.5.2. Risk Stratification and Antimicrobial Policy

Interleukin-10 has been investigated as a potentially valuable biomarker for risk stratification in pediatric febrile neutropenia, particularly in identifying children at elevated risk for bacteremia and severe infections. Doerflinger et al. (2021) [16] demonstrated that a dual-biomarker model combining IL-10 ≥ 4.37 pg/mL and procalcitonin ≥ 0.425 ng/mL achieved 100% sensitivity (95% CI: 68.8–100%) and 89% specificity (95% CI: 80.0–95.0%) for bacteremia prediction. This approach correctly identified all eight bacteremia episodes, classified 20% of cases as high risk (PPV = 50%), and excluded 80% as low risk (NPV = 100%), thereby complementing clinical decision criteria. Similarly, Xu et al. (2019) [27] evaluated IL-10 for early detection of septic shock, reporting that levels ≥ 20 pg/mL at fever onset yielded an AUC of 0.87 (95% CI: 0.83–0.90), with 77% sensitivity and 79% specificity. IL-10 outperformed CRP (AUC = 0.65) and PCT (AUC = 0.78), although IL-6 demonstrated slightly better discrimination (AUC = 0.89). Elevated IL-10 independently predicted septic shock (OR = 4.28, 95% CI: 2.56–7.15; *p* < 0.001) and identified 87% of shock cases among bacteremic patients. Araujo et al. (2017) [3] further supported IL-10’s diagnostic relevance, showing that IL-10 > 6 pg/mL at fever onset achieved 69% sensitivity and 86% specificity (AUC = 0.83, NPV = 83%, PPV = 75%) for sepsis discrimination. Septic patients exhibited 4.5-fold higher median IL-10 levels (8.5 vs. 1.9 pg/mL; *p* = 0.004), with a marked decline by Day 5 (1.1 pg/mL; *p* = 0.007). Notably, filgrastim administration increased median IL-10 (5.51 vs. 1.2 pg/mL; *p* = 0.004) without compromising diagnostic performance (AUC remained ≥ 0.83). Combining IL-10 with PCT > 180 pg/mL improved specificity to 91%. The rapid decline in IL-10 (5-fold by Day 2 in bacteremia cases) demonstrated the biomarker’s limited kinetic utility, as shown by Doerflinger et al. (2021) [16], who emphasized the superiority of PCT’s increasing pattern over decreasing cytokine levels. Xia et al. (2016) [35] confirmed IL-10’s predictive capacity for severe infections, reporting an AUC of 0.839 (95% CI: 0.806–0.880), with high specificity (94.9%) but modest sensitivity (47.1%) at a 100 pg/mL threshold. IL-6 displayed marginally better discrimination (AUC = 0.875). Both cytokines outperformed PCT (AUC = 0.726) and CRP (AUC = 0.648), supporting their utility for early intervention. Adjusting the IL-10 cutoff to 49.5 pg/mL improved sensitivity to 57.1%, though specificity decreased to 91.1%, illustrating a clear threshold dependence.

Indeed, reported IL-10 cutoffs in pediatric FN range widely—from 4.37 pg/mL to 100 pg/mL—with lower thresholds (e.g., 4.37 pg/mL) favoring sensitivity for bacteremia detection when combined with PCT, and higher thresholds (e.g., 100 pg/mL) offering better specificity for severe infections. This variability likely stems from divergent clinical endpoints (e.g., bacteremia vs. septic shock vs. MODS), differing assay platforms (e.g., ELISA vs. Luminex), and inherent limitations in sample sizes across studies. These factors underscore the need for site-specific validation of IL-10 thresholds and highlight the importance of combining IL-10 with complementary biomarkers (e.g., PCT, IL-6) and consistent timing of sample collection relative to fever onset.

Urbonas et al. (2012) [17] demonstrated that IL-10 < 18 pg/mL had a negative predictive value of 83% for excluding bacteremia/sepsis in pediatric FN, with 92% specificity (PPV = 86%, AUC = 0.87) and a sensitivity CI ranging from 39% to 94%. This supports its inclusion in risk stratification algorithms for this high-risk population. Likewise, Şahbudak Bal et al. (2017) [32] reported that IL-10 < 5.04 pg/mL yielded a 95.2% NPV (sensitivity = 92.9%, specificity = 44.4%) for excluding culture-confirmed bacteremia or fungemia. While affirming IL-10’s role in risk stratification, the authors emphasized IL-8’s superior NPV of 97.4% for excluding Gram-negative infections—suggesting its value as a complementary biomarker in multimodal diagnostic frameworks. The authors called for larger validation cohorts.

In summary, IL-10 demonstrates robust diagnostic potential in pediatric FN when integrated into multimodal biomarker strategies. Its ability to stratify risk early in the febrile episode—particularly in conjunction with PCT—supports its inclusion in diagnostic algorithms. However, its performance remains threshold- and timing-dependent, necessitating rigorous local validation for clinical application.

### 2.6. Additional Biomarkers

Among the evaluated cytokines, several—including G-CSF, IL-1β, TNF-α, soluble TNF receptor-II (sTNFR-II), IL-2, IL-22, IL-12/23p40, IL-17, and MCP-1α—have demonstrated limited or inconsistent diagnostic utility in pediatric febrile neutropenia. Their clinical value appears significantly inferior to established biomarkers such as IL-6, IL-10, IL-8, and PCT, particularly when assessed as standalone indicators.

**IL-1β and TNF-α** have been investigated for sepsis detection in febrile neutropenia, but consistent evidence from prospective and longitudinal studies has shown their limited diagnostic and prognostic utility. Soker et al. (2001) [18] established this pattern, finding no significant differences in IL-1β (undetectable in most cases) or TNF-α levels (median: 7.8–8.4 pg/mL; *p* > 0.05) between culture-positive and culture-negative FN episodes, though TNF-α tended to be higher in Gram-negative infections (13.1 vs. 6.3 pg/mL; *p* = 0.07). Riikonen et al. (1992) [19] corroborated these findings, reporting elevated TNF-α levels (>40 ng/L) in almost all febrile neutropenic children but no significant distinction between bacteremic and non-bacteremic episodes. TNF-α elevations were nonspecific, observed uniformly across viral, fungal, and bacterial infections. While IL-6 showed weak correlation with CRP during Days 1–2 of fever (*r* = 0.25, *p* = 0.02), serum amyloid A demonstrated superior sensitivity for bacteremia (100% vs. CRP’s 68%)—though the study cautioned against its standalone use due to false-positives in viral infections. Notably, the original study did not report AUC values for SAA or CRP, and TNF-α levels lacked correlation with CRP. These results underscore TNF-α’s limited diagnostic utility in FN. Xu et al. (2019) [27], in a prospective study of 3118 febrile episodes in pediatric cancer patients, confirmed TNF-α’s limited discriminative capacity. While TNF-α levels were significantly higher in bloodstream infections (median: 2.7 vs. 2.3 pg/mL; *p* < 0.001) and septic shock (3.0 vs. 2.3 pg/mL; *p* < 0.001), its diagnostic performance remained poor (AUC = 0.651, 95% CI: 0.594–0.707 for septic shock), vastly inferior to IL-6 (AUC = 0.888, 95% CI: 0.861–0.914) and IL-10 (AUC = 0.866, 95% CI: 0.828–0.903). TNF-α’s clinical utility was further undermined by its low elevation rate (10.2% of episodes vs. 85.2% for IL-6) and poor specificity. The study concluded that TNF-α serves better as a severity marker than a diagnostic tool, particularly when compared to IL-6 and IL-10, which outperformed CRP and IFN-γ (AUC = 0.574 for septic shock). Abrahamsson et al. (1997) [12] demonstrated impaired TNF-α production in children receiving chemotherapy compared to those without chemotherapy (14.0 vs. 37.7 pg/mL; *p* < 0.001), with neutropenic patients showing significantly lower TNF-α levels than non-neutropenic patients (*p* < 0.001). However, TNF-α showed no significant differences across bacteremic, FUO, and non-bacterial episodes (*p* > 0.05), with elevation in only 27% of septic patients (9/34). IL-6 showed 74% sensitivity and 49% specificity for bacteremia, with 45% positive predictive value, but correlated poorly with early CRP (*r* = 0.26 on day 1 vs. 0.53 on day 2). Despite IL-6’s superior sensitivity compared to CRP (74% vs. 29%), its elevation in 57% of FUO episodes and failure to detect 26% of bacteremia precluded standalone use for antibiotic decisions, underscoring the limitations of single biomarkers in neutropenic fever management. Hatzistilianou et al. (2010) [7] evaluated the diagnostic performance of cytokines in 94 children with ALL across different neutropenic and infectious states. TNF-α levels were elevated in bacterial infections (46.1 pg/mL in neutropenic vs. 82.4 pg/mL in non-neutropenic patients) but demonstrated suboptimal diagnostic performance (AUC = 0.777) compared to procalcitonin (AUC = 0.875, sensitivity 94%, specificity 96.5%). IL-1β showed the poorest discrimination among cytokines (AUC = 0.616), while IL-8 (AUC = 0.750) performed better than IL-1β but remained inferior to PCT. The study concluded that PCT was superior to all tested cytokines (TNF-α, IL-1β, IL-8) for distinguishing between bacterial and viral infections in this population. Kharya et al. (2007) [10] evaluated biomarkers in 62 febrile neutropenic episodes, demonstrating TNF-α’s inability to distinguish septic from non-septic cases (*p* > 0.05). IL-6 showed superior diagnostic performance, with AUCs of 0.73 (admission) and 0.77 (48 h). A 20 pg/mL IL-6 threshold achieved 100% sensitivity, capturing all sepsis cases (15/15) and fatalities (6/6). PCT showed 73% sensitivity at >1.73 ng/mL but missed fungal infections, while hsCRP showed no discrimination. The study proposed IL-6 < 20 pg/mL + PCT < 1 ng/mL as criteria for identifying low-risk patients, confirming TNF-α’s limited clinical utility. Aggarwal et al. (2013) [15] evaluated 52 febrile neutropenia episodes, demonstrating TNF-α’s nonspecific elevation in high-risk infections (41.6 vs. 22.4 pg/mL; *p* = 0.38) with poor specificity (35% at 5 pg/mL cutoff). Despite high NPV (88%), TNF-α—like IL-6 (NPV = 82%) and IL-8 (NPV = 88%)—failed to differentiate infection severity (*p* > 0.05 for all). IL-5 levels (4.0–4.5 pg/mL) were clinically irrelevant (*p* = 0.26). Only 7/52 episodes had microbiologically confirmed infections (*E. coli*, *S. aureus*), underscoring cytokines’ role as exclusion tools (NPV > 80%) rather than diagnostic discriminators. Xia et al. (2016) [35] analyzed 320 blood culture-positive sepsis episodes in pediatric hematology/oncology patients, revealing TNF-α’s poor diagnostic performance. Only 18.1% (58/320) of sepsis cases showed TNF-α elevations above the normal range, while TNF-α levels in viral infections remained low (median 2.7 pg/mL vs 60.1 pg/mL in bacteremia), demonstrating its poor discriminatory ability between infectious etiologies Median TNF-α levels were 60.1 pg/mL in severe sepsis (shock/MODS) versus 7.0 pg/mL in mild infections, demonstrating significant overlap. Notably, CRP’s AUC for Gram-negative bacteremia was just 0.596 (95% CI: 0.527–0.666)—worse than IL-10 (AUC = 0.747) and PCT (AUC = 0.657). TNF-α showed poor diagnostic performance, with only an 18.1% positivity rate in bacteremia, though specific AUC values for TNF-α were not reported for Gram-negative cases. The study concluded TNF-α lacked specificity for bacterial etiology and could not discriminate infection severity, reinforcing its limited clinical utility compared to IL-6/IL-10.

Longitudinal analyses reinforced these conclusions. Kim et al. (2022) [26] tracked 10 pediatric bacteremia episodes, observing rapid declines in IL-6 (*p* < 0.001) and IL-10 (*p* = 0.001) by Day 4, while TNF-α showed no significant changes (*p* > 0.05). These kinetics were independent of fever duration (IL-6/IL-10: *p* = 1.000 for ≥3 vs. <3 days). CRP and 11 other cytokines also showed no fever-associated patterns. The study concluded cytokine kinetics reflect immune restoration rather than drive prolonged fever. Similarly, Araujo et al. (2017) [3] tracked cytokine kinetics over 14 days in 35 febrile neutropenic children, finding TNF-α levels nonspecific (1.44 vs. 4.6 pg/mL septic/non-septic; *p* = 0.16) and IL-1β undetectable in >90% of cases. IL-6 (AUC = 0.87), IL-8 (AUC = 0.86), and IL-10 (AUC = 0.83) demonstrated superior diagnostic performance as sepsis discriminators, unlike TNF-α, which showed no significant differences between groups (*p* = 0.16), with rapid declines by Day 3 (*p* < 0.005). The IL-23/IL-17 axis was deficient in septic patients (*p* < 0.05), and filgrastim upregulated IL-6/IL-8/IL-10 (*p* < 0.05). PCT (AUC = 0.89) was the strongest discriminator and was included in the study’s main conclusions as a useful early biomarker. Cost et al. (2013) [29] analyzed 195 febrile neutropenia episodes, demonstrating TNF-α’s poor discriminatory power in univariate analysis (54.3 vs. 38.0 pg/mL; *p* = 0.56), leading to its exclusion from the final multivariate model, despite stepwise significance due to minimal added predictive value. IL-8 dominated predictions (AUC = 0.81; OR = 6.06), with a cutoff of <1260 pg/mL achieving 90% sensitivity and 98% NPV. Two false-negative cases (pre-B ALL in maintenance) occurred. The study reinforced TNF-α’s nonspecificity and IL-8’s utility for risk stratification, aligning with the prior literature

**Soluble TNF receptor-II (sTNFR-II)** Soluble TNF receptor-II (sTNFR-II) has consistently demonstrated limited diagnostic and prognostic utility in FN, as evidenced by complementary findings from Karakurt et al. (2014) [30] and Hatzistilianou et al. (2010) [7]. Karakurt et al. (2014) [30] evaluated 50 episodes of febrile neutropenia in pediatric cancer patients and found that soluble TNF receptor II (sTNFR-II) levels showed no significant differences across infection types: microbiologically confirmed (510.28 pg/mL), clinically documented (874.50 pg/mL), and fever of unknown origin (949.43 pg/mL; *p* = 0.30). In univariate analysis, higher sTNFR-II levels were associated with fever lasting more than 3 days (674.03 vs. 423.61 pg/mL; *p* = 0.04), but this association lost statistical significance in multivariate analysis (*p* = 0.051). IL-6 (*p* = 0.012) and IL-8 (*p* = 0.003) emerged as independent predictors of fever duration. The empirical monotherapy with piperacillin/tazobactam achieved 58% success without treatment modification. The study concluded that sTNFR-II likely reflects a nonspecific inflammatory response, while IL-6 and IL-8 are more reliable biomarkers for risk stratification in febrile neutropenia. In a related study, Hatzistilianou et al. (2010) [7] investigated 94 children with acute lymphoblastic leukemia (ALL) during febrile episodes, assessing the diagnostic utility of soluble TNF receptor II (sTNFR-II) and other cytokines. sTNFR-II levels were elevated in bacterial infections—5666 pg/mL in neutropenic and 3887 pg/mL in non-neutropenic patients. The diagnostic performance of sTNFR-II was moderate (AUC = 0.795), ranking second among cytokines tested but still inferior to procalcitonin (PCT, AUC = 0.875). sTNFR-II outperformed TNF-α (AUC = 0.777) and IL-8 (AUC = 0.750) in diagnostic accuracy. However, the authors found sTNFR-II unsuitable for monitoring treatment response due to a high percentage of persistently elevated levels during therapy, contrasting with PCT’s ability to reflect clinical improvement through declining levels.

Together, these studies solidify sTNFR-II’s role as a nonspecific marker of inflammatory burden rather than a reliable tool for infection classification or outcome prediction in FN. While transient elevations may reflect prolonged febrile episodes, its overlap across etiologies and poor multivariate performance against IL-6/IL-8 render it clinically unactionable.

**IL-2 and IL-22**, cytokines involved in T-cell activation and mucosal immunity, have been proposed as potential biomarkers for infection subtype differentiation, though results remain inconclusive. These cytokines demonstrated time-dependent associations with bacterial etiology in a small exploratory study by Kim et al. (2022) [26] involving 10 pediatric bacteremia episodes, IL-2 and IL-22 exhibited distinct temporal patterns associated with bacterial etiology. IL-2 levels were transiently elevated in Gram-positive infections on Day 1 (median: 6.05 pg/mL vs. 3.00 pg/mL in Gram-negative; *p* = 0.038), while IL-22 showed a delayed increase in Gram-negative infections by Day 8 (76.46 pg/mL vs. 47.32 pg/mL in Gram-positive; *p* = 0.010). However, these differences were not sustained at other timepoints (Days 4 and 8 for IL-2; Days 1 and 4 for IL-22), and their statistical significance was limited by the lack of correction for multiple comparisons across the 13 cytokines analyzed at three timepoints. The cohort’s extreme heterogeneity—including diverse underlying malignancies, chemotherapy regimens, and infection sites (respiratory vs. gastrointestinal)—further complicated the interpretation of these findings. The authors emphasized these observations’ potential for hypothesis generation while noting the study’s limitations, including the inability to establish definitive clinical utility for pathogen differentiation based on this preliminary data. Cost et al. (2013) [29] confirmed the limited diagnostic utility of IL-2 in their prospective study of 195 febrile neutropenia episodes. Although IL-2 was measured alongside nine other cytokines, no significant association was found between IL-2 levels and bacteremia (median: 2.8 pg/mL in bacteremic vs. 0.1 pg/mL in non-bacteremic episodes; *p* = 0.37). IL-22 was not included in their cytokine panel (which comprised IL-1β, IL-2, IL-4, IL-5, IL-6, IL-8, IL-10, IFN-γ, TNF-α, and GM-CSF), underscoring its lower relevance in early biomarker studies. The considerable heterogeneity of the cohort—spanning various malignancies, chemotherapy protocols, and infection sites—further complicated data interpretation. While the authors viewed their findings as hypothesis generating, they concluded that IL-2 lacked clinical value for infection differentiation. In contrast, IL-8 remained the strongest predictor in multivariate models (AUC = 0.81), while IL-2 and TNF-α were excluded because they did not improve model performance.

Xia et al. (2016) [35], in their analysis of 320 culture-positive sepsis episodes among 2819 febrile episodes, found no significant differences in median IL-2 levels across diagnostic groups: bacteremic (3.3 pg/mL), clinically diagnosed sepsis (3.2 pg/mL), and non-sepsis infections (3.1 pg/mL; all *p* > 0.05). IL-2 was therefore not considered diagnostically useful. In contrast, IL-6 and IL-10 demonstrated superior discriminative power, with AUC values exceeding 0.8 for predicting severe infection.

**IL-12/23p40 and IL-17** were analyzed in the context of sepsis biomarkers, revealing crucial findings about mucosal barrier integrity. Araujo et al. (2017) [3] found that IL-17 and IL-12/23p40 levels were significantly lower in septic neutropenic children (2.22 vs. 26.5 pg/mL; *p* = 0.04 and 93.5 vs. 263.3 pg/mL; *p* = 0.044, respectively). A strong linear correlation between the two cytokines (R^2^ = 0.88) suggested disruption of the IL-23–Th17 axis. These cytokines were excluded from ROC analysis due to their limited diagnostic performance, with the study focusing instead on IL-6, IL-8, and IL-10 (AUC > 0.8). The axis impairment was attributed to chemotherapy-induced lymphocyte depletion and was observed predominantly in patients with gut-derived bacteremia (five out of eight cases), pointing to mucosal barrier breakdown as a plausible pathophysiological mechanism.

**Monocyte chemoattractant protein-1-alpha (MCP-1α)**, a chemokine involved in monocyte recruitment, demonstrated strong specificity (92.3%) and positive predictive value (95%) for infection detection in febrile neutropenic children (El-Maghraby et al., 2007 [42]). However, 35.6% of infected patients (21/59) had MCP-1α levels below the diagnostic cutoff, including several with mild infections and coagulase-negative staphylococcal bacteremia. While CRP levels correlated specifically with Gram-negative bacteremia (*p* = 0.034), MCP-1α lacked pathogen-type specificity (*p* = 0.277). A composite model incorporating CRP, IL-8, and MCP-1α significantly enhanced diagnostic performance, with at least two markers elevated in 78% of infected cases versus only 16% in non-infected cases (*p* < 0.001). These findings support the role of MCP-1α in multimarker diagnostic strategies rather than as a standalone indicator.

**Soluble interleukin-2 receptor (sIL-2R)** has been proposed for risk prediction in febrile neutropenia. Soluble interleukin-2 receptor (sIL-2R) demonstrated 77% sensitivity and 81% negative predictive value for bacteremia/sepsis detection in pediatric oncology patients at 1658 U/mL cutoff, with moderate specificity (67%). Urbonas et al. (2013) [22] found that combining PCT with sIL-2R yielded an AUC of 0.82, slightly higher than PCT alone (AUC = 0.79). Both biomarkers showed significantly elevated levels in bacteremia/sepsis compared to fever of unknown origin episodes (*p* < 0.001 for PCT, *p* = 0.0024 for sIL-2R). PCT demonstrated superior diagnostic performance, outperforming sIL-2R in specificity (92% vs. 78% at diagnostic cutoffs) and positive predictive value (82% vs. 63%). While sIL-2R showed good screening characteristics with 85% sensitivity and 84% NPV at the 1558 ng/L cutoff, it provided no additional diagnostic advantage when combined with PCT, leading the authors to conclude that PCT alone is sufficient for clinical decision-making in pediatric febrile neutropenia. Karakurt et al. (2014) [30] reported median sIL-2R levels of 12.07 ng/mL (range: 3.09–42.58) in 50 febrile neutropenia episodes, with no significant differences between lymphoproliferative (17.46 ng/mL) and solid tumors (13.83 ng/mL; *p* = 0.06) or infection types (microbiological: 24.75 ng/mL; clinical: 14.46 ng/mL; *p* = 0.34). Univariate analysis showed higher sIL-2R in fever > 3 days (19.20 vs. 11.14 ng/mL; *p* = 0.04), but multivariate analysis confirmed no independent predictive value for hospitalization (*p* = 0.092), antibiotic duration (*p* = 0.966), or neutropenia recovery (*p* = 0.887). Unlike IL-6/IL-8 (*p* = 0.012/0.003 for fever duration), sIL-2R was a secondary marker with limited prognostic utility. Soker et al. (2001) [18] observed significantly higher sIL-2R in Gram-negative (5244 U/mL, range: 2290–7600; *n* = 7) vs. Gram-positive bacteremia (1480 U/mL, range: 1120–2105; *n* = 4; *p* = 0.006) in pediatric cancer patients with neutropenia, excluding two fungal cases. The authors suggested that elevated sIL-2R, along with IL-6 and IL-8, may guide empirical antibiotic initiation while awaiting culture results. Notably, sIL-2R showed complete separation between Gram-negative and Gram-positive infections with no overlapping ranges, suggesting strong discriminatory potential. However, they emphasized that small sample sizes limit generalizability, and findings require validation in larger cohorts before routine clinical application.

**Presepsin** (soluble CD14 subtype, sCD14-ST), a 13 kDa fragment cleaved from monocyte/macrophage surface marker CD14 during bacterial phagocytosis, has emerged as a promising biomarker for early infection detection [67]. Unlike traditional markers, presepsin levels rise within 2 h of inflammation onset, preceding increases in procalcitonin and CRP [68]. In a prospective study conducted by Baraka and Zakaria (2018) [41], 60 pediatric patients with hematological malignancies and febrile neutropenia were evaluated for presepsin, CRP, and PCT levels. Presepsin concentrations were significantly elevated in patients with bacteremia compared to those with clinically documented infections or fever of unknown origin, with reported diagnostic sensitivity and specificity of 100% and 85.7%, respectively. These findings supported presepsin’s superior diagnostic performance relative to PCT and CRP in detecting bloodstream infections in this vulnerable population. Similarly, the study by Arikan et al. (2021) [44] included 47 febrile neutropenia episodes in 39 children with hematological malignancies. Serial measurements of presepsin were performed at admission (day 0), and on days 3 and 7 of antibiotic treatment, showing higher levels in bacteremic episodes at all time points, though statistical significance was not achieved (*p* = 0.10, *p* = 0.67, *p* = 0.75 respectively). The combined use of presepsin with CRP and/or PCT improved the predictive capacity for bacteremia, as confirmed by multivariate logistic regression and ROC analysis. Cerasi et al. (2023) [43] observed that while presepsin levels were significantly elevated in febrile neutropenic patients compared to healthy controls, no significant difference was found between bacteremic and non-bacteremic cases either at fever onset or 48 h later. Notably, presepsin levels at 48 h post-fever onset were higher in patients who developed unfavorable outcomes, including ICU admission or septic shock, suggesting a potential prognostic rather than diagnostic role in this setting. In a single-center prospective cohort of 36 febrile neutropenia episodes in 26 pediatric oncology patients, Agnello et al. (2020) [46] assessed serial presepsin and midregional proadrenomedullin (mr-proADM) levels at admission (T0), Day 2 (T1), and Day 5 (T2). Both markers were elevated at T0 and declined progressively by T2. Notably, presepsin levels differed significantly between bacteremia and fever of unknown origin (FUO) groups only at T1 (median: 416 vs. 271 pg/mL, *p* = 0.021), with low diagnostic accuracy (AUC 0.58). Similarly, mr-proADM showed poor discriminatory capacity (AUC 0.62). Despite limited diagnostic performance, both markers were significant predictors of hospitalization duration at T0 (presepsin: *p* = 0.00007; mr-proADM: *p* = 0.0038), supporting their potential prognostic value rather than their utility for early infection stratification.

**G-CSF** is a well-established mediator of granulopoiesis and neutrophil mobilization. Produced in response to inflammatory stimuli—especially bacterial infections—G-CSF enhances phagocytic activity and supports acute host defense. Although not essential under steady-state conditions, its production by alveolar macrophages during infection is critical for neutrophil recruitment and activation [69,70]. Beyond innate immunity, G-CSF influences adaptive responses by suppressing T-cell proliferation and promoting the differentiation of regulatory dendritic cells, contributing to immune tolerance [71]. As a biomarker, G-CSF has shown early elevation in neonatal and pediatric sepsis, with more pronounced levels in bacterial versus viral infections [72,73].

In earlier work by Bux et al. (1999) [21] demonstrated infection-specific G-CSF elevation (60–1006 pg/mL) in 20/63 antibody-induced neutropenia patients with active infections (e.g., otitis media, staphylodermia), while non-infected patients maintained normal levels (<39 pg/mL). In contrast, eight severe congenital neutropenia (SCN) patients showed constitutively high G-CSF (85–1800 pg/mL) irrespective of infection, attributed to defective granulopoiesis and chronic bacterial colonization. This dichotomy supports G-CSF’s role in distinguishing infectious (acute cytokine-driven) from non-infectious neutropenic states, though SCN’s small sample size warrants caution in generalization. Tapia et al. (2021) [6] reported a 14.6-fold median elevation in G-CSF levels in microbiologically confirmed infections (7742.5 vs. 530.44 pg/mL in FN-UO; *p* < 0.001). ROC analysis showed G-CSF alone had AUC = 0.763, improving to 0.839 (75% sensitivity, 81% specificity) when combined with IL-6, Flt-3L, and IP-10. Comparative analysis between bacterial and viral infections revealed significantly higher G-CSF levels in bacterial infections, with AUC = 0.665 for bacterial detection. The combined model leveraged G-CSF’s bacterial discrimination capability and IP-10’s viral specificity (AUC = 0.702 for viral detection).

Together, these findings highlight G-CSF’s context-dependent diagnostic utility—rising acutely in response to infection in immune-mediated neutropenia and febrile episodes, while remaining elevated in non-infectious congenital neutropenia. Its integration into multiplex biomarker models enhances discriminatory accuracy, particularly for differentiating bacterial from viral etiologies.

**Pancreatic stone protein (PSP)**, initially characterized as lithostathine, is a 14 kDa glycoprotein secreted by pancreatic acinar cells, later identified as a systemic stress marker with immunomodulatory properties. PSP shares structural similarity with C-type lectin-like proteins and is upregulated in response to tissue injury, inflammation, and infection. It is secreted not only in pancreatic disorders but also in response to remote organ damage, suggesting a role in systemic immune responses. These features have led to its investigation as a promising biomarker for infection and sepsis across various clinical contexts [74]. In a prospective study by Antari et al. (2023) [45], PSP levels were evaluated in 70 episodes of febrile neutropenia in pediatric patients with hematological malignancies. PSP concentrations on Day 1 were significantly higher in children with sepsis compared to those without (179 ng/mL vs. 80 ng/mL, *p* < 0.00001). PSP demonstrated good diagnostic accuracy for sepsis with a sensitivity of 84% and specificity of 82%, outperforming both MR-proADM (Mid-Regional pro-Adrenomedullin) and CRP. Additionally, PSP levels were significantly elevated in patients with bloodstream infections (*p* = 0.03), supporting its potential utility in early risk assessment and infection characterization in immunocompromised pediatric populations.

**Mid-Regional Proadrenomedullin** is a stable peptide fragment derived from the precursor molecule adrenomedullin (ADM), a multifunctional vasodilatory peptide belonging to the calcitonin gene-related peptide family. ADM is involved in endothelial homeostasis, immune modulation, and antimicrobial defense. MR-proADM serves as a surrogate marker for ADM levels due to its greater plasma stability and longer half-life. In the context of febrile neutropenia, MR-proADM has shown promising utility in identifying children at increased risk of severe outcomes, including sepsis and septic shock [75]. In the study by Antari et al. (2023) [45], MR-proADM levels were significantly elevated in septic versus non-septic pediatric FN patients (0.559 vs. 0.196 nmol/L, *p* = 0.02), although its diagnostic performance (AUC = 0.68, Se = 74%, Sp = 70%) was lower than that of PSP (AUC = 0.80). Notably, MR-proADM demonstrated a distinct kinetic pattern with progressive elevation over time (day 1: 0.314 nmol/L; day 7: 0.609 nmol/L), contrasting with the declining trend observed for PSP and CRP. Given its mechanistic association with endothelial dysfunction and vascular integrity during severe infection, MR-proADM may serve as a complementary biomarker in the assessment of immunocompromised children, particularly for monitoring disease progression rather than early detection. In support of this, Agnello et al. (2020) [46] observed that MR-proADM levels were elevated at admission and predicted hospitalization length, although their diagnostic accuracy for infection etiology was limited. Consistent with these findings, Ragab et al. (2025) [47] reported that MR-proADM demonstrated excellent diagnostic accuracy for identifying bacterial infections in pediatric patients with febrile neutropenia, with an AUC of 0.964, sensitivity of 90.5%, and specificity of 82.6% at a cutoff value of 489 pg/mL. Moreover, MR-proADM levels were significantly higher in patients with bacteremia and those who experienced severe clinical courses, including prolonged fever duration and extended hospitalization. Importantly, MR-proADM showed stronger correlation with hospital stay duration than either CRP or PCT (*r* = 0.838, *p* < 0.001), further underscoring its prognostic value. These results reinforce the potential role of MR-proADM as a reliable biomarker for risk stratification in immunocompromised pediatric populations. Similarly, Fawzi et al. (2019) [48] investigated MR-proADM in a cohort of 100 pediatric patients with hematologic malignancies and severe neutropenia. Their study demonstrated that MR-proADM was significantly elevated on the first day of fever in patients with bacteremia or sepsis, compared to those with pyrexia of unknown origin. Receiver operating characteristic (ROC) analysis yielded an AUC of 0.939 for MR-proADM on Day 1—substantially outperforming CRP (AUC = 0.509). At a cutoff value of 2.4 nmol/L, MR-proADM achieved a sensitivity of 91.6% and specificity of 85.1%, further supporting its diagnostic utility in the early phase of infection. These findings emphasize that MR-proADM can serve as a superior early biomarker compared to CRP, which failed to significantly differentiate between infectious and non-infectious febrile episodes in this context. Although Fawzi et al. did not evaluate long-term prognostic outcomes, their results reinforce the value of MR-proADM in early clinical decision-making in neutropenic pediatric populations. In contrast, Susanto et al. (2022) [49] reported more limited diagnostic performance of MR-proADM in a cohort of 60 pediatric patients undergoing cancer-related chemotherapy. Using a cutoff value of 2.88 nmol/L, the biomarker yielded an AUC of 0.707, with sensitivity of 60.0% and specificity of 56.67%. Interestingly, MR-proADM levels were paradoxically higher in the non-sepsis group (median 2.51 nmol/L) than in the sepsis group (median 0.194 nmol/L), a finding that deviates from most other reports. Although the difference was statistically significant (*p* = 0.006), the overall diagnostic accuracy (58.33%) was modest. The authors concluded that MR-proADM alone may not reliably distinguish sepsis in this clinical context and emphasized the need for combining biomarker results with clinical assessment to enhance diagnostic precision. Supporting this diagnostic potential, Arıkan et al. (2021) [44] evaluated MR-proADM in 47 febrile neutropenia episodes among 39 pediatric patients with hematological malignancies. The study demonstrated that MR-proADM levels were significantly elevated in FN patients with bacteremia compared to healthy controls (58.24 vs. 46.13 pg/mL, *p* < 0.001). Using a cutoff of 57.95 pg/mL, the biomarker yielded an AUC of 0.78 for diagnosing FN episodes (distinguishing FN patients from healthy controls), with a sensitivity of 98% and specificity of 75%. However, no significant difference was observed in MR-proADM levels between bacteremic and non-bacteremic FN episodes across all time points (*p* = 0.90, 0.67, and 0.70 for Days 1, 3, and 7, respectively), nor between Gram-positive and Gram-negative bacteremia (*p* = 0.93, 0.99, 0.68), indicating its limitations in differentiating bacterial etiology within FN episodes. The authors suggested that serum proADM levels may alert physicians to bacteremic episodes in pediatric FN patients, but emphasized that further studies are needed to verify or disprove this hypothesis In a related study. Demirkaya et al. (2015) [50] explored the diagnostic value of adrenomedullin (ADM) in 50 febrile neutropenia episodes among 37 pediatric oncology patients. The researchers observed that ADM levels on Day 3 were significantly higher in patients with microbiologically documented infections (MDI) compared to those with clinically documented infections (CDI) and fever of unknown origin (FUO), with median values of 191.7 pg/mL (range: 32.1–695.4) for MDI, 42.8 pg/mL (21.2–110.1) for CDI, and 46.0 pg/mL (23.3–91.3) for FUO. Notably, no significant difference was found between the MDI and sepsis groups (58.7 pg/mL, range: 19.7–100.1). Although the study did not establish a diagnostic cutoff, these results suggest that ADM may help identify microbiologically confirmed infections in the subacute phase of febrile neutropenia. However, its ability to differentiate sepsis or predict clinical deterioration appeared limited, reinforcing the view that the unstable kinetics of native ADM may reduce its clinical utility compared to the more stable MR-proADM fragment. Accordingly, the authors recommended that future research focus on the application of MR-proADM as a potentially more reliable biomarker in this population.

## 3. Summary

Pooled evidence from 46 pediatric studies (Table 1) confirms that IL-6 and procalcitonin remain the most validated biomarkers for early infection detection in pediatric febrile neutropenia, though their clinical utility depends on rigorous contextual interpretation. IL-6 demonstrates robust diagnostic performance for bacteremia (AUC 0.867; Kitanovski 2006 [11]) and septic shock (AUC 0.89; Xu 2019 [27]), outperforming C-reactive protein, which shows moderate specificity (76.6% at >40 mg/L; Santolaya 1994 [23]). PCT achieves comparable discrimination (AUC 0.875–0.92; Hatzistilianou et al. (2010) [7], Baraka 2018 [41]), with specificity ranging from 29.4% (Purkayastha 2016 [40]) to 100% at high cutoffs (Akcay 2021 [31]), necessitating threshold-dependent interpretation.

IL-10 provides reliable bacteremia exclusion when combined with PCT (89% specificity, 100% NPV), while PCT alone achieved AUC 0.842 and IL-10 alone AUC 0.826 (Doerflinger 2021 [16].) IL-8 effectively stratifies low-risk episodes, achieving an NPV of 100% at ≤60 ng/L with normal vital signs (Nijhuis 2005 [34]) and 98% at <1260 pg/mL for low-risk bacteremia (Cost 2013 [29]). CRP retains perfect sensitivity for culture-proven infection (100% at >40 mg/L; Santolaya 1994 [23]) but exhibits reduced specificity in leukemia cohorts (66.7% at ≥105 mg/L; Baraka 2018 [41]).

Emerging biomarkers show population-specific utility. Presepsin has shown promising diagnostic potential in pediatric febrile neutropenia, with Baraka and Zakaria (2018) [41] reporting high sensitivity (100%) and specificity (85.7%) for bacteremia. However, Arikan et al. (2021) [44] found that presepsin levels were not significantly elevated in bacteremic episodes when used alone (*p* = 0.10, 0.67, 0.75 for days 1, 3, and 7, respectively) but showed improved diagnostic performance when combined with CRP and PCT in multivariate analysis. However, Cerasi et al. (2023) [43] found no significant difference in presepsin levels between bacteremic and non-bacteremic patients at fever onset, suggesting limited diagnostic utility in certain clinical contexts, though its prognostic value for unfavorable outcomes at 48 h was noted.

PSP demonstrated strong diagnostic performance (AUC = 0.8), with elevated levels in sepsis and bloodstream infections in pediatric febrile neutropenia (Antari 2023 [45]). Similarly, MR-proADM showed excellent diagnostic accuracy (AUC = 0.964) and strong prognostic potential, with significant correlations with clinical severity and hospital stay duration (Ragab 2025 [47]). However, some studies (e.g., Susanto 2022 [49]) report inconsistent MR-proADM performance, and ADM itself lacks sufficient discriminatory value (Demirkaya 2015 [50]). Thus, while both markers may enhance early risk stratification, further multicenter validation is warranted. Multicenter trials addressing these limitations are imperative before integrating novel markers into clinical algorithms.

G-CSF demonstrated AUC = 0.763 for distinguishing infectious from non-infectious episodes, while IP-10 showed AUC = 0.702 specifically for viral infection detection, both validated in persistent high-risk febrile neutropenia (Tapia 2021 [6]). In contrast, sIL-2R displays only moderate performance (AUC 0.73; Urbonas 2013 [22], contradicting earlier claims of Gram-negative specificity (Soker 2001 [18]).

## 4. Discussion

Recent advances in immunodiagnostics have enabled the exploration of cytokine and acute-phase protein profiles as tools for improving infection detection and risk stratification in pediatric patients with chemotherapy-induced neutropenia. Multiple studies have demonstrated that biomarkers such as IL-6, PCT, IL-10, and IL-8 offer superior sensitivity and specificity compared to conventional markers like CRP, particularly in the early phase of infection and in differentiating between bacterial and viral etiologies. However, despite this progress, several pediatric-specific challenges remain that must be addressed before routine clinical implementation.

Pooled evidence from 46 pediatric studies (Table 1) confirms that IL-6, Il-8, procalcitonin, and CRP remain the most validated biomarkers for early infection detection in pediatric febrile neutropenia, though their clinical utility depends on rigorous contextual interpretation. IL-6 demonstrates robust diagnostic performance for bacteremia (AUC 0.867; Kitanovski 2006 [11]) and septic shock (AUC 0.89; Xu 2019 [27]), outperforming C-reactive protein, which shows moderate specificity (76.6% at >40 mg/L; Santolaya 1994 [23]). PCT achieves comparable discrimination (AUC 0.875–0.92; Hatzistilianou et al. (2010) [7], Baraka 2018 [41]), with specificity ranging from 29.4% (Purkayastha 2016 [40]) to 100% at high cutoffs (Akcay 2021 [31]), necessitating threshold-dependent interpretation.

IL-10 provides reliable bacteremia exclusion when combined with PCT (89% specificity, 100% NPV; Doerflinger 2021 [16]), with PCT achieving AUC 0.842 and IL-10 AUC 0.826 as standalone biomarkers (Doerflinger 2021 [16]). IL-8 effectively stratifies low-risk episodes, achieving an NPV of 100% at ≤60 ng/L with normal vital signs (Nijhuis 2005 [34]) and 98% at <1260 pg/mL for low-risk bacteremia (Cost 2013 [29]). CRP retains perfect sensitivity for culture-proven infection (100% at >40 mg/L; Santolaya 1994 [23]) but exhibits reduced specificity in leukemia cohorts (66.7% at ≥105 mg/L; Baraka 2018 [41]).

Emerging biomarkers show population-specific utility. While presepsin demonstrated high diagnostic performance in some cohorts (Baraka 2018 [41]), its accuracy varied significantly across studies, with several recent investigations, including Arikan 2021 [44] and Cerasi et al. (2023) [43], reporting limited discrimination of bacteremia at fever onset (*p* = 0.989; AUC 0.508). G-CSF demonstrated AUC = 0.763 for distinguishing infectious from non-infectious episodes, while IP-10 showed AUC = 0.702 specifically for viral infection detection, both validated in persistent high-risk febrile neutropenia (Tapia 2021 [6]) In contrast, sIL-2R displays only moderate performance (AUC 0.73; Urbonas 2013 [22]), contradicting earlier claims of Gram-negative specificity (Soker 2001 [18]). PSP and MR-proADM have emerged as promising biomarkers for sepsis discrimination. In pediatric febrile neutropenia, PSP demonstrated superior diagnostic performance (AUC = 0.80) compared to CRP (AUC = 0.67), while MR-proADM showed comparable performance (AUC = 0.68) (Antari 2023 [45]). In a separate study, MR-proADM achieved excellent diagnostic accuracy (AUC = 0.964) for predicting bacterial infections in similar patient populations (Ragab 2025 [47]). Their diagnostic utility shows promise, though inter-study variability (e.g., Susanto 2022 [49] with paradoxical results) highlights the need for validation before establishing prognostic relevance.

Critical evidence gaps remain. Filgrastim upregulates IL-6 and IL-8 levels in febrile neutropenia (Araújo 2017 [3]), but this pharmacodynamic effect does not compromise their diagnostic utility for sepsis. Karakurt (2014) [30] documented a high prevalence of therapeutic confounders (growth factors in 72% episodes, recent chemotherapy in all patients) but did not analyze their effects on biomarker dynamics, representing a potential limitation in cytokine interpretation. Multicenter trials addressing these limitations are imperative before integrating novel markers into clinical algorithms.

Among the markers studied, IL-6 has demonstrated the most consistent diagnostic and prognostic utility, often outperforming CRP and PCT. Its rapid response to bacterial infection and association with septic complications make it a leading candidate for early identification of high-risk patients. Similarly, PCT provides a dynamic tool for infection monitoring and antibiotic stewardship, with high specificity for bacterial etiologies and declining levels correlating with clinical improvement. IL-10, while primarily anti-inflammatory, has shown value as a “rule-out” marker due to its high specificity and negative predictive value for bacteremia and sepsis. IL-8, especially when used in conjunction with other cytokines, offers strong discriminatory power for infection severity and has potential in identifying low-risk patients.

Emerging markers such as G-CSF, TNF, presepsin, PSP, MR-proADM, sIL-2R, sTNFR2, Flt3L, and IP-10 are beginning to define more nuanced immune profiles associated with infection type and severity. These may eventually serve as components of multiplex biomarker panels or support machine-learning-based risk models that integrate cytokine dynamics with clinical and microbiological data.

**Figure 1:**
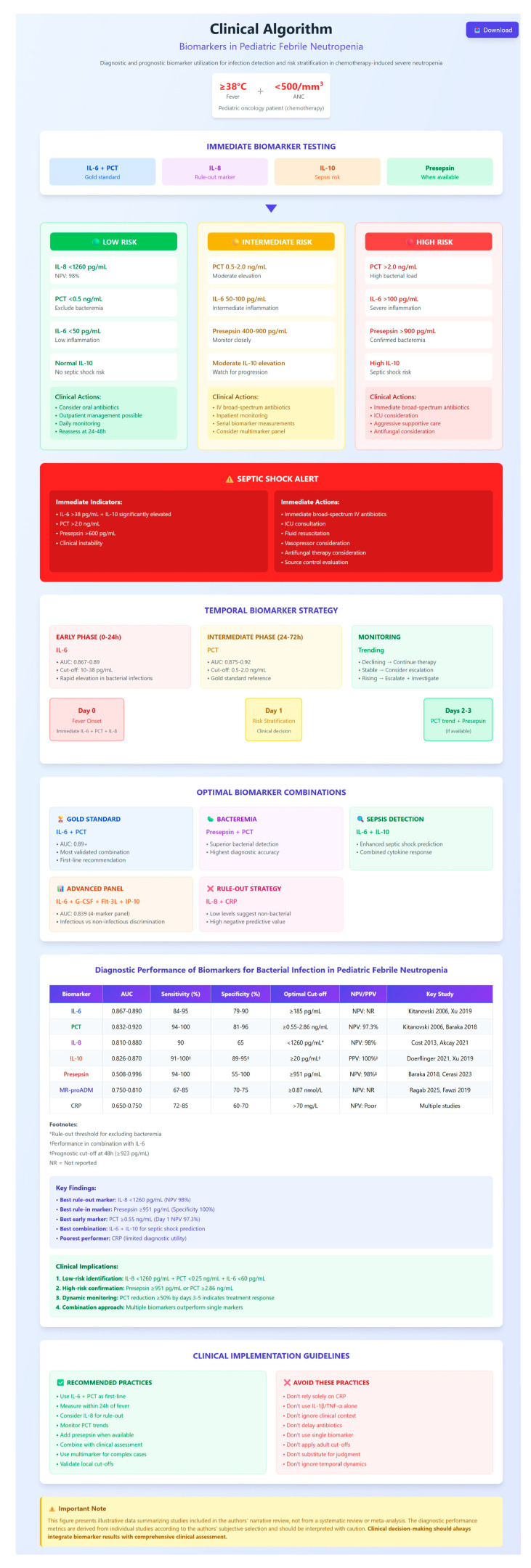
Clinical algorithm for biomarkers in pediatric febrile neutropenia. Proposed approach for diagnostic and prognostic biomarker utilization in infection detection and risk stratification during chemotherapy-induced severe neutropenia. The algorithm incorporates immediate biomarker testing (IL-6, PCT, IL-8, IL-10, Presepsin), three-tier risk stratification (low/intermediate/high risk), septic shock alert criteria, optimal biomarker combinations, and temporal monitoring strategy. The accompanying table summarizes diagnostic performance metrics (AUC, sensitivity, specificity, optimal cutoffs) for key biomarkers based on pooled evidence from pediatric studies. Important Note: This figure presents illustrative data summarizing studies included in the authors’ narrative review, not derived from a systematic review or meta-analysis. The diagnostic performance metrics represent individual study findings according to the authors’ subjective selection and should be interpreted with caution. This algorithm is intended for educational purposes and is not meant to replace clinical judgment or serve as formal clinical recommendations. Clinical decision-making should always integrate biomarker results with comprehensive clinical assessment, patient-specific factors, and institutional protocols.

The aim of this review was to present recent research findings and research directions regarding biomarkers in FN. Although our analysis did not constitute a formal meta-analysis or systematic review, we aimed to summarize the obtained results regarding biomarker utilization in pediatric FN and demonstrate their potential clinical applications. Therefore, Figure 1 presents an infographic with our proposed utilization for risk stratification of FN episodes, along with a table of diagnostic performance metrics for the most important markers. Given that this analysis was not conducted systematically and represents merely a straightforward summary of the authors’ subjective selection, it should be interpreted only as illustrative and was in no way prepared as clinical recommendations.

A critical factor in interpreting cytokine data in children is the age-dependent development of the immune system, which differs substantially from that in adults. The pediatric immune response is characterized by evolving balances between innate and adaptive immunity, as well as age-related shifts in cytokine production. For instance, neonates and infants may exhibit lower baseline pro-inflammatory responses or delayed cytokine kinetics compared to older children and adolescents [72,73]. This developmental trajectory affects physiological cytokine levels, making direct extrapolation of adult-derived thresholds inappropriate and potentially misleading. Moreover, immunological immaturity may lead to atypical infection presentations or blunted inflammatory responses, especially in children undergoing intensive chemotherapy.

One of the major obstacles in translating cytokine biomarkers into practice is the absence of standardized pediatric reference intervals. Most studies rely on locally derived or adult-based thresholds, which may not account for interindividual variability, underlying disease, or treatment phase. The lack of harmonization across assay platforms, sample handling procedures, and cytokine quantification methods further complicates interpretation. This variability has resulted in heterogeneous cutoffs for the same biomarker across different studies, limiting the ability to establish universal clinical decision points. Standardization initiatives, ideally incorporating age-specific normal ranges and harmonized assay protocols, are essential for meaningful comparisons and broader clinical applicability.

Pediatric cancers are rare, with many classified as ultra-rare diseases, significantly limiting the feasibility of large, prospective, and statistically powered biomarker studies. As a result, much of the available data are derived from single-center studies with relatively small cohorts, which, while informative, carry limitations in generalizability and reproducibility. Multi-institutional collaborations and data sharing efforts are urgently needed to consolidate findings, validate proposed biomarker thresholds, and construct robust predictive models. Additionally, heterogeneous treatment regimens and supportive care protocols across centers may introduce further variability in cytokine expression profiles.

Importantly, infectious complications remain a major contributor to treatment failure in pediatric oncology, independent of the malignancy’s inherent resistance. While advances in supportive care and antimicrobial prophylaxis have improved outcomes, a significant proportion of mortality still results from sepsis, septic shock, and infection-associated organ failure during periods of profound neutropenia. Therefore, improving early infection recognition and enhancing risk stratification through biomarkers is not a peripheral issue—it is central to the overarching goal of reducing preventable mortality in pediatric cancer care.

Nevertheless, several limitations persist. The clinical utility of cytokines is currently constrained by limited assay availability in routine practice, the high cost of multiplex panels, and the need for rapid turnaround times in acute care settings. Furthermore, many cytokines exhibit pleiotropic behavior, and their levels can be influenced by non-infectious inflammation, treatment-related toxicity, or concurrent use of agents like G-CSF and corticosteroids.

The current evidence base underscores the significant potential of cytokines and related biomarkers in improving diagnostic precision and personalizing infection management in febrile neutropenic children. However, for these biomarkers to move from research to routine care, several critical steps are needed:

- establishment of pediatric-specific reference values and cutoffs;
- standardization of assay methodologies and reporting formats;
- development of cost-effective, rapid platforms suitable for real-time decision-making;
- and validation through prospective, multicenter trials with unified protocols.

However, it is important to emphasize that the present review is not intended as a meta-analysis nor does it aim to establish clinical recommendations for specific biomarker applications. The primary objective was to provide a structured overview of the current research landscape and to summarize the reported diagnostic performance of the most frequently investigated biomarkers in pediatric febrile neutropenia. Given the substantial heterogeneity in study designs, populations, timepoints, and reporting formats, we intentionally refrained from suggesting direct clinical applicability of individual markers or combinations.

Incorporating cytokine profiles into risk-adapted treatment algorithms holds promise not only for reducing infection-related morbidity and mortality, but also for minimizing overtreatment and optimizing resource use. Ultimately, precision diagnostics in pediatric febrile neutropenia will require a thoughtful integration of clinical judgment, microbiological data, and immunological biomarkers tailored to the unique physiology of children.

## Figures and Tables

**Table 1:** Summarization of the key studies evaluating diagnostic biomarkers in febrile neutropenic children.

No.	First Author (Year) [Citation No.]	Markers Studied	Population Studied	Marker(s) Results and Cutoff	Diagnostic Insights from This Study	Prognostic Value	Cytokine/Other Marker in Distinguishing Infection Etiology	Integrating Cytokine/Other Marker in Risk Stratification
1	Tapia (2021) [6]	IL-6, G-CSF, Flt-3L, IP-10 (plus 34 others)	110 pediatric cancer patients with persistent high-risk febrile neutropenia (HRFN); multicenter study, Chile	IL-6 significantly elevated on day 4 in patients with FN-DEA vs. FN-UO: median 80.81 vs. 13.37 pg/mL; *p* = 0.002. IL-6: AUC = 0.701; G-CSF: AUC = 0.763; Flt-3L: AUC = 0.745. IP-10: AUC = 0.702 for viral infections. Combined G-CSF, Flt-3L, IL-6, and IP-10: AUC = 0.839, sensitivity 75%, specificity 81%.	On day 4 of persistent HRFN, cytokine/chemokine levels were significantly higher in episodes with identified infectious agent (FN-DEA), indicating a measurable immune response.	IL-6 and G-CSF were significantly elevated in FN-DEA. Two of three fatal cases occurred in FN-DEA group, suggesting potential prognostic relevance, although not statistically analyzed.	G-CSF discriminated bacterial infections (AUC = 0.665); IP-10 identified viral infections (AUC = 0.702). Flt-3L and IL-6 also showed diagnostic power.	Authors propose using combined levels of G-CSF, IL-6, Flt-3L and IP-10 on day 4 of HRFN to help differentiate infectious from non-infectious causes; AUC = 0.839.
2	Hatzistilianou (2010) [7]	PCT, CRP, TNF-α, IL-1β, IL-8, sTNFRII	54 febrile episodes in neutropenic children with ALL (group A); comparison with non-neutropenic and non-bacterial groups,	PCT > 2 ng/mL predicted bacterial infection in neutropenic patients with sensitivity 94% and specificity 96.5%; AUC = 0.875. IL-1β AUC = 0.616; IL-8 AUC = 0.750; TNF-α AUC = 0.777; sTNFRII AUC = 0.795.	PCT levels elevated early in bacterial infection and decreased with response to therapy; best single discriminator of bacterial infection on day 1 compared to other markers	PCT decrease during treatment paralleled clinical improvement and successful antibiotic response; persistently high levels linked with prolonged or complicated infections	PCT discriminated well between bacterial and viral infections; IL-8 specificity was high in afebrile controls but limited in bacterial vs. viral discrimination. other markers (CRP, TNF-α, IL-1β, sTNFRII) were less effective	PCT proposed as valuable tool for early prediction of bacterial infections and monitoring therapy in febrile neutropenic children with ALL; no formal risk stratification model proposed
3	Nahar (2023) [8]	Procalcitonin (PCT), C-reactive protein (CRP)	58 children with acute leukemia and febrile neutropenia, ages 1 < 18 years; single center, Bangladesh	Median PCT in bacteremia: 26.10 μg/L vs. 0.78 μg/L (no bacteremia), *p* = 0.002. PCT > 2 μg/L significantly associated with bacteremia. AUC: PCT = 0.797 (95% CI 0.651–0.943); CRP = 0.697 (95% CI 0.54–0.855). Median CRP: 137.4 mg/L (bacteremia) vs. 54.17 mg/L (no bacteremia), *p* = 0.036.	PCT and CRP measured within 24 h of FN onset; both useful in early detection of bacterial infection, PCT superior to CRP based on AUC.	not applicable	PCT > 2 μg/L significantly associated with bacteremia. PCT better discriminator than CRP based on ROC curve.	not applicable
4	Martinez-Albarran (2009) [9]	Procalcitonin (PCT), C-reactive protein (CRP)	54 pediatric patients with febrile neutropenia and cancer, Mexico; prospective observational study	PCT (cutoff 0.67 ng/mL): sensitivity 72.2%, specificity 80.5%. CRP (cutoff 9.06 mg/dL): sensitivity 77.7%, specificity 72.2%. Median PCT: high-risk 3.2 ng/mL vs. low-risk 1.39 ng/mL (*p* = 0.003); CRP: high-risk 16.28 mg/dL vs. low-risk 6.78 mg/dL (*p* = 0.001)	PCT and CRP significantly higher in patients classified as high-risk (systemic infection or sepsis). PCT more specific; CRP more sensitive. Both useful for early identification of infection.	Mortality occurred only in high-risk group (4/18), but difference in marker levels between deceased and survivors with complications was not statistically significant	PCT and CRP both distinguished high- vs. low-risk infection. PCT had higher specificity; CRP had higher sensitivity. No data on organism-specific discrimination.	Authors recommend using PCT and/or CRP in risk group classification at FN onset. No formal algorithm provided.
5	Kharya (2007) [10]	IL-6, hsCRP, TNF-α, PCT	62 febrile neutropenia episodes in children (5 months–17 years) with malignancy/aplastic anemia; India	IL-6 at 0 h > 137.5 pg/mL: sensitivity 67%, specificity 75%. IL-6 at 48 h > 69 pg/mL: sensitivity 75%, specificity 78%. PCT > 1.73 ng/mL at 0 h: sensitivity 73%, specificity 70%. IL-6 > 20 pg/mL: 100% sensitivity for sepsis (all 15 proven cases). PCT > 1 ng/mL: 80% sensitivity (12/15 proven cases).	IL-6 and PCT significantly elevated in sepsis vs. non-sepsis. IL-6 at admission and 48 h had AUC 0.73 and 0.77 respectively. Both useful for early identification.	All deaths (*n* = 6) occurred in patients with IL-6 > 20 pg/mL. Elevated IL-6 and PCT associated with proven sepsis and poor outcome.	IL-6 > 20 pg/mL and PCT > 1 ng/mL included all proven sepsis cases. Lower IL-6 values reliably excluded sepsis.	Authors suggest IL-6 and PCT could guide early discharge decisions or withholding antibiotics in low-risk patients (e.g., IL-6 < 20 pg/mL, PCT < 1 ng/mL).
6	Kitanovski (2006) [11]	IL-6, PCT, CRP	68 febrile neutropenia episodes in 32 pediatric cancer patients (hematologic malignancies or solid tumors); Slovenia	IL-6 day 1 > 235.1 pg/mL **: AUC 0.867, sensitivity 87.5%, specificity 86.0%. PCT day 1 > 0.55 μg/L **: AUC 0.832, sensitivity 93.8%, specificity 70.6%. CRP day 1 > 60 mg/L **: AUC 0.649, sensitivity 62.5%, specificity 70.0%.	IL-6 and PCT significantly elevated in bacteremia/clinical sepsis vs. localized infection and FUO; better early markers than CRP. Sequential PCT improved diagnostic accuracy.	not applicable	No statistically significant differences in IL-6 or PCT based on pathogen type. CRP had lower initial accuracy. IL-6 and PCT better for early detection of bacteremia/sepsis.	not applicable
7	Abrahamsson (1997) [12]	IL-6, TNF-α, IFN-γ, CRP	110 febrile episodes in 70 children with malignancy (hematologic and solid tumors), Sweden	IL-6 ≥ 50 pg/mL: sensitivity 74%, specificity 49% in detecting bacteremia. CRP ≥ 50 mg/L: sensitivity 29%, specificity 78%.	IL-6 more sensitive than CRP in detecting bacterial infection in neutropenic and non-neutropenic patients. Elevated IL-6 also seen in FUO.	not applicable	IL-6 levels higher in sepsis vs. NBF (*p* < 0.05). No significant difference in IL-6 between sepsis and FUO. TNF-α and IFN-γ levels less useful.	not applicable
8	Srinivasan (2021) [13]	Procalcitonin (PCT), IL-6, IL-8	46 febrile neutropenia episodes in 33 children with cancer; prospective study, India	PCT ≥ 2 ng/mL: sensitivity 63%, specificity 91%, NPV 88%, PPV 70%; AUC = 0.745. IL-6 (cutoff 50 pg/mL): sensitivity 54%, specificity 57%, NPV 80%, PPV 28%; AUC = 0.574. IL-8 (cutoff 130 pg/mL): sensitivity 45%, specificity 49%, NPV 73%, PPV 21%; AUC = 0.551.	PCT, IL-6, and IL-8 measured at admission. PCT showed best diagnostic performance for documented infections. IL-6 and IL-8 had lower accuracy, but favorable NPV.	not applicable	PCT better predictor of bacteremia than IL-6 and IL-8. No significant differences in IL-6/IL-8 between infected and non-infected groups.	PCT, IL-6, and IL-8 may help define low-risk patients suitable for early discharge and outpatient treatment. No formal model presented.
9	Diepold (2008) [14]	IL-6, IL-8, CRP	123 febrile neutropenic episodes in 69 pediatric oncology patients undergoing chemotherapy; Germany	IL-6 > 42 pg/mL: sensitivity 90%, specificity 85%, PPV 94%, NPV 77%. IL-8 > 30 pg/mL: sensitivity 87%, specificity 59%, PPV 84%. CRP > 1 mg/dL: sensitivity 83%, specificity 59%, PPV 86%.	IL-6 and IL-8 measured within 24 h of fever onset. IL-6 best predictor of sepsis or prolonged fever. IL-6 > 42 pg/mL identifies high-risk patients; low levels suggest short fever duration.	not applicable	IL-6 and IL-8 higher in sepsis and prolonged fever vs short fever. IL-6 better predictor than IL-8 or CRP. No differences between Gram-positive and Gram-negative noted.	IL-6 < 42 pg/mL may help define low-risk patients for early discharge or outpatient therapy; model not formally validated.
10	Aggarwal (2013) [15]	IL-5, IL-6, IL-8, TNF-α	52 episodes of febrile neutropenia in 48 children with hematologic malignancies; India	IL-6 > 100 pg/mL: sensitivity 36%, specificity 24%, NPV 59%. IL-8 > 300 pg/mL: sensitivity 46%, specificity 26%, NPV 59%. TNF-α > 25 pg/mL: sensitivity 71%, specificity 33%, NPV 88%. IL-5: not useful due to narrow range.	IL-5, IL-6, IL-8, TNF-α measured within 24 h of admission. None of the cytokines could predict high-risk FN, but IL-6, IL-8, TNF-α had high NPV (>80%) for ruling out serious infection.	not applicable	IL-6, IL-8 and TNF-α not significantly different between low- and high-risk groups; however, their high NPV suggests utility in excluding serious infection.	not applicable
11	Doerflinger (2021) [16]	PCT, IL-10, MIP-1β, CRP, IL-6, IL-8 (plus 28 others)	79 febrile neutropenia episodes in 64 children with cancer; multicenter study, Australia	PCT: ≥0.425 ng/mL (sensitivity 100%, specificity 78%, AUC = 0.842). IL-10: ≥16.66 pg/mL (sensitivity 75%, specificity 83%, AUC = 0.826). Combined PCT + IL-10: ≥0.425 ng/mL + ≥4.37 pg/mL (sensitivity 100%, specificity 89%). MIP-1β: AUC = 0.780 (*p* = 0.010). CRP/IL-6/IL-8: Non-significant (AUC = 0.695/0.625/0.666; *p* > 0.05).	PCT + IL-10 best predicted bacteremia at fever onset. CRP/IL-6/IL-8 had no discriminatory value. Day 2: PCT rose 11-fold in bacteremia cases.	not applicable	PCT + IL-10 distinguished bacteremia; IL-6/IL-8 did not.	PCT + IL-10 improved AUS-rule CDR performance (84% low-risk classification vs. 73% with AUS-rule alone); supports early discharge decisions.
12	Urbonas (2012) [17]	IL-10	36 febrile neutropenia episodes in 24 pediatric oncology patients (19 hematologic [ALL, AML, NHL], 5 solid tumors); Lithuania	IL-10 ≥ 18 pg/mL: sensitivity 73%, specificity 92%, PPV 86%, NPV 83%; AUC = 0.87	IL-10 significantly higher in sepsis vs. FUO group (median 39 vs. 0 pg/mL, *p* = 0.0006). High specificity and NPV support IL-10 as a tool to exclude infection early.	not applicable	IL-10 significantly higher in SEP vs FUO; no overlap in medians. Effective in distinguishing sepsis/bacteremia from non-infectious fever.	Rule-out tool for sepsis/bacteremia (high NPV/specificity). Not a standalone predictor (no formal CDR model)
13	Soker (2001) [18]	IL-1β, sIL-2R, IL-6, IL-8, TNF-α	48 febrile neutropenia episodes in 23 pediatric cancer patients (ALL, AML, NHL); Turkey	IL-6, sIL-2R, and IL-8 significantly higher in culture-positive vs. culture-negative and control groups (*p* < 0.001). IL-6: 57 pg/mL vs. 8.6 pg/mL vs. 4.0 pg/mL; IL-8: 305 pg/mL vs. 23 pg/mL vs. 5 pg/mL. IL-1β and TNF-α not significantly different.	IL-6, IL-8, sIL-2R elevated early in febrile neutropenia, especially in culture-positive episodes. May guide empirical antibiotic initiation.	not applicable	IL-6, IL-8, sIL-2R significantly higher in Gram-negative vs. Gram-positive bacteremia (*p* = 0.042, 0.023, 0.006). IL-1β and TNF-α not useful in identifying infection type.	not applicable
14	Riikonen (1992) [19]	TNF-α, IL-1β, IL-6, CRP, SAA	81 febrile episodes in 46 neutropenic children with cancer; Finland	TNF-α > 40 ng/L in most patients; range up to 520 ng/L. IL-1β > 20 ng/L in all groups. IL-6 detectable in 68% of episodes, with elevated concentrations in 15%. SAA > 5 mg/L in all bacteremia cases (mean normal = 2.1 ± 3.2 mg/L). CRP < 20 mg/L in 32% of bacteremia episodes. CRP and SAA correlated (r = 0.63, *p* < 0.001).	All markers elevated in fever, but none differentiated bacteremia from other causes. IL-6 correlated with CRP on days 1 and 2. SAA more sensitive than CRP for early detection of bacteremia.	not applicable	No cytokine or acute-phase protein clearly differentiated between bacteremia, FUO, focal or non-bacterial infection.	not applicable
15	Miedema (2010) [20]	IL-8, PCT, CRP, sTREM-1	43 febrile neutropenic episodes in 29 children with cancer; Netherlands; prospective study	IL-8 (cutoff 60 ng/L): AUC = 0.81 (95% CI: 0.656–0.965), sensitivity 92%, specificity 54% PCT (cutoff 0.25 ng/mL): AUC = 0.84 (95% CI: 0.710–0.970), sensitivity 79%, specificity 77%, significant predictor at 24–48 h (*p* = 0.047) CRP (cutoff 40 mg/L): AUC = 0.60 (95% CI: 0.388–0.803), sensitivity 69%, specificity 62% (*p* = 0.183) IL-8 + PCT combination: 100% sensitivity, 52% specificity for MDI	IL-8 and PCT measured at admission. Both markers performed well in early detection of microbiologically documented infection (MDI).	not applicable	IL-8 and PCT significantly higher in MDI vs. no infection. IL-8 median: 318.5 vs. 55 ng/L (*p* = 0.002); PCT: 0.81 vs. 0.10 ng/mL (*p* = 0.001).	IL-8 + PCT combination showed potential for risk stratification; 100% sensitivity in identifying MDI.
16	Bux (1999) [21]	G-CSF	63 patients with antibody-induced neutropenia (including neonatal, autoimmune, and drug-induced forms)	G-CSF elevated (60–1006 pg/mL) only in patients with concurrent infections; normal < 39 pg/mL G-CSF levels decreased to normal after successful antibiotic treatment”	Elevated G-CSF levels observed only in presence of infection, not due to neutropenia itself	not applicable	not applicable	not applicable
17	Urbonas (2013) [22]	PCT, sIL-2R, sHLA-G, presepsin	62 febrile neutropenia episodes in 37 pediatric oncology patients (ALL, AML, NHL, solid tumors); Lithuania	PCT: AUC = 0.79, optimal cutoff 0.38 ng/mL; diagnostic cutoff 0.65 ng/mL: specificity 92%, PPV 82%. sIL-2R: AUC = 0.73; screening cutoff 1558 ng/L: sensitivity 85%, specificity 56%. Presepsin: median 401 vs. 356 ng/L (*p* = 0.82); sHLA-G: *p* = 0.07	PCT and sIL-2R significantly higher in BS vs FUO group. sHLA-G showed a non-significant trend (*p* = 0.07); presepsin not useful diagnostically.	not applicable	PCT and sIL-2R helped distinguish bacteremia/sepsis from FUO; sHLA-G and presepsin did not.	Combined PCT + sIL-2R AUC = 0.82, but no improvement over PCT alone (AUC = 0.79); PCT recommended as primary marker.
18	Santolaya (1994) [23]	CRP	85 febrile neutropenia episodes in 75 pediatric cancer patients (ALL, lymphoma, solid tumors); Chile. Group I = confirmed bacterial infection (culture-positive); Group II = probable bacterial infection (severe clinical/radiologic findings, culture-negative); Group III = viral or no infection.	CRP > 40 mg/L: sensitivity 100%, specificity 76.6% for demonstrated bacterial infection (day 1); mean CRP: group I = 194 mg/L, group II = 143 mg/L, group III = 29 mg/L; *p* < 0.001	CRP > 40 mg/L on day 1 significantly associated with bacterial infection; CRP useful for early diagnosis and monitoring	Persistently high CRP associated with poor outcomes; patients with unfavorable course had non-decreasing CRP	CRP > 40 mg/L on day 1 discriminated bacterial from viral/no infection; *p* < 0.001	Authors propose using day 1–2 CRP levels (>40 mg/L) to guide antibiotic initiation; CRP decline supports treatment efficacy
19	Jaing (2020) [24]	IL-8, CRP	30 febrile neutropenic pediatric patients after allogeneic HSCT; Taiwan. Group I = unexplained fever; Group II = clinically/radiologically documented infection.	IL-8 ≥ 60 ng/L and CRP ≥ 40 mg/L associated with documented infection; IL-8 decreased earlier than CRP; IL-8 and CRP positively correlated (*r* = 0.289, *p* = 0.039)	IL-8 elevated earlier than CRP; both markers higher in infection group; CRP significantly higher in group II (*p* < 0.05); IL-8 not significantly different	not applicable	IL-8 and CRP useful in distinguishing unexplained fever from documented infectious episodes	IL-8 < 60 ng/L (2 consecutive values) used to identify low-risk patients eligible for antibiotic de-escalation
20	Urbonas (2011) [25]	IL-8	16 pediatric oncology patients with febrile neutropenia and bloodstream infections (11 Gram-negative, 5 Gram-positive); Lithuania	IL-8 measured on day 1 and day 2 of FN. Median IL-8 values in Gram-negative infection group were 3.9–4.3 times higher than in Gram-positive infection group. Specific values or cutoff not provided. Mann–Whitney test used for comparison.	Distinct cytokine response to Gram-negative vs. Gram-positive bacteria; IL-8 elevated significantly more in Gram-negative infections on day 1–2	not applicable	IL-8 levels significantly higher in Gram-negative bloodstream infections (3.9–4.3×, *p* < 0.05).	Proposed as adjunct diagnostic tool for early bacteremia typing (days 1–2), but requires validation in larger cohorts. Not yet validated for risk stratification.
21	Kim (2022) [26]	IL-2, IL-4, IL-5, IL-6, IL-9, IL-10, IL-13, IL-17A, IL-17F, IL-21, IL-22, TNF-α, IFN-γ	10 pediatric patients (3–18 yrs) with febrile neutropenia and bacteremia during chemotherapy or HCT for hematologic malignancies; Korea	IL-6 and IL-10: highest on Day 1 (median IL-6: 429.68 pg/mL (73.07–8030.00)), significantly decreased by Day 4 (*p* < 0.001 and *p* = 0.001, respectively); IL-2 higher in GPB vs. GNB on Day 1 (6.05 vs. 3.00 pg/mL, *p* = 0.038); IL-22 higher in GNB vs. GPB on Day 8 (76.46 vs. 47.32 pg/mL, *p* = 0.010); no consistent cutoff values reported	IL-6 and IL-10 concentrations were significantly elevated at onset of FN and declined within 4 days regardless of fever duration; IL-2 and IL-22 showed statistical differences depending on bacterial type (GPB vs. GNB)	No severe complications (shock, ARDS, death); cytokine kinetics not associated with prolonged fever ≥3 days; all patients survived except two who died due to leukemia progression	IL-2 (Day 1) higher in GPB; IL-22 (Day 8) higher in GNB; other cytokines not discriminatory; potential for IL-2/IL-22 in early etiology differentiation	Cytokine kinetics (esp. IL-6/IL-10) peaked early and normalized by Day 4; no link to fever duration; authors conclude immune-modulating therapy not supported for prolonged fever
22	Xu (2019) [27]	CRP, PCT, IL-6, IL-10, TNF-α, IFN-γ	3118 febrile illness episodes in 1115 pediatric cancer patients (mainly ALL, AML, lymphoma); China	For GNB: IL-10 AUC = 0.81 (cutoff 18.5 pg/mL), IL-6 AUC = 0.77 (cutoff 185 pg/mL), PCT AUC = 0.68, CRP AUC = 0.56. For septic shock: IL-6 AUC = 0.89 (cutoff 185 pg/mL, Se 84.4%, Sp 79.3%), IL-10 AUC = 0.87 (cutoff 20 pg/mL), PCT AUC = 0.78, CRP AUC = 0.65	IL-6 and IL-10 were significantly elevated in GNB and septic shock; better predictors than CRP or PCT; CRP not independently predictive	IL-6 ≥ 185 pg/mL (OR 8.21) and IL-10 ≥ 20 pg/mL (OR 4.28) independently predicted septic shock; 12 deaths recorded (8 with shock)	IL-6 and IL-10 identified GNB and septic shock more accurately than CRP/PCT; IL-10 ≥ 18.5 pg/mL and IL-6 ≥ 185 pg/mL discriminated severe infection	IL-6 < 185 pg/mL and IL-10 < 20 pg/mL combination identified low-risk patients (shock rate 0.7%); proposed for early risk stratification
23	van der Galiën (2018) [28]	IL-6, PCT	77 febrile neutropenia episodes in 55 children with cancer (hematologic, solid, brain tumors); Netherlands	IL-6 cutoff = 60 ng/L: AUC T0 = 0.88, Se 100%, Sp 34.7%, NPV 100%; IL-6 T1: AUC = 0.86, Se 100%, Sp 54.2%; PCT cutoff = 0.25 ng/mL: AUC T0 = 0.70, Se 93.3%, Sp 42.8%; PCT T1: AUC = 0.77, Se 90%, Sp 37.1%	IL-6 and PCT significantly higher in bacterial vs non-bacterial episodes; IL-6 more sensitive than PCT; combining both improved accuracy	not applicable	IL-6 and PCT (individually and combined) were significantly elevated in bacterial infection; IL-6 alone identified all bacterial cases	IL-6 > 60 and/or PCT > 0.25 ng/mL at admission: sensitivity 100%, NPV 100%; Combined rule (IL-6 ≥ 60 ng/L or PCT ≥ 0.25 ng/mL) achieved 100% sensitivity/NPV; the inverse rule (both biomarkers below cutoffs) identified 41% as low-risk
24	Cost (2013) [29]	IL-8 (plus IL-1β, IL-2, IL-4, IL-5, IL-6, IL-10, TNF-α, IFN-γ, GM-CSF), CRP	195 febrile neutropenia episodes in 116 pediatric oncology patients (chemotherapy or radiotherapy; no HSCT); single center, USA	IL-8 < 1260 pg/mL predicted low risk for bacteremia: sensitivity 90%, specificity 65%, NPV 98%, PPV 25%; AUC = 0.81; IL-8 levels significantly higher in bacteremia vs. non-bacteremia (median 4479 vs. 553 pg/mL, *p* < 0.001)	IL-8 most predictive marker in multivariate model; outperformed clinical predictors and other cytokines; proposed as single-variable risk stratification tool	2 deaths due to bacteremia (Pseudomonas + Enterococcus, and S. pneumoniae);	IL-8 significantly elevated in bacteremia vs non-bacteremia; not influenced by concurrent respiratory viral infection	IL-8 < 1260 pg/mL identified low-risk patients with 98% NPV; authors propose IL-8-based triage to reduce unnecessary hospitalizations
25	Karakurt (2014) [30]	IL-6, IL-8, sTNFRII, sIL-2R, CRP, PCT	50 febrile neutropenia episodes in 31 pediatric cancer patients (non-leukemic); Turkey	IL-6: median 94.7 pg/mL (6.7–1729.9), IL-8: 125.9 pg/mL (18.2–3362.4), sTNFRII: 628.7 pg/mL, sIL-2R: 12.1 ng/mL, CRP: 1.8 mg/dL. IL-6, IL-8, sTNFRII significantly higher in fever >3 days (*p* = 0.02, 0.01, 0.04). IL-8 higher in treatment modification group (*p* = 0.048). PCT ≥ 1 ng/mL associated with prolonged fever (*p* = 0.014). No cutoff values defined.	IL-6, IL-8, sTNFRII correlated with prolonged fever; IL-8 also associated with treatment modification; no single marker predictive of severe infectious complications	not applicable	IL-6, IL-8, sTNFRII levels higher in microbiologically proven infections; CRP paradoxically lower in infection vs. FUO; differences not statistically significant	IL-6, IL-8, sTNFRII suggested as supportive markers in early identification of prolonged febrile course; authors recommend cautious use in clinical decisions
26	Akcay (2021) [31]	IL-6, IL-8, MBL, CRP, PCT	54 febrile neutropenic episodes in 30 pediatric cancer patients (ALL, AML, NHL, neuroblastoma); Turkey	IL-8 ≥ 200 pg/mL: sensitivity 80%, specificity 65%, NPV 92%; IL-6 ≥ 100 pg/mL: sensitivity 80%, specificity 40%, NPV 88%; PCT ≥ 10 ng/mL: specificity 100%, PPV 100%, NPV 84%; CRP not discriminatory; MBL ≥ 400 ng/mL: sensitivity 80%, specificity 59%, NPV 93%	IL-8 significantly higher in B/S vs. CMDI (*p* = 0.038) and FUO (*p* = 0.012); IL-6, PCT, MBL highest in B/S but without statistical significance; CRP not useful diagnostically	not applicable	IL-8 was best marker for distinguishing B/S from CMDI/FUO; other markers showed no significant differences between infection types	IL-8 ≥ 200 pg/mL had the highest discriminatory power and 92% NPV for B/S, but its standalone use is limited by 65% specificity. The study advocates combining it with PCT (specificity 100%) for risk stratification.; proposed as part of early low-risk identification strategy
27	Şahbudak Bal (2017) [32]	IL-6, IL-8, IL-10	59 febrile neutropenia episodes in 38 pediatric patients with ALL or AML; prospective study, Turkey	IL-8 ≥ 61.3 pg/mL: Sens. 64.1%, Spec. 75.6%, PPV 45%, NPV 87.2%; IL-10 ≥ 5.04 pg/mL: Sens. 92.9%, Spec. 44.4%, PPV 34.2%, NPV 95.2%; IL-6 ≥ 98.8 pg/mL: Sens. 50%, Spec. 71.1%, PPV 35%, NPV 81.1%	IL-6, IL-8, and IL-10 levels were significantly higher during infection. IL-10 was the most sensitive and IL-8 the most specific for predicting culture-confirmed infection.	not applicable	IL-8 showed the highest specificity and NPV for Gram-negative bacteremia, with AUC = 0.772 (*p* = 0.008); IL-10 had the highest sensitivity for overall culture-confirmed infections, AUC = 0.725 (*p* = 0.003).	IL-8 may be useful in ruling out Gram-negative infections; IL-10 may be used to rule out bacterial infection in general due to high NPV.
28	Araujo (2017) [3]	IL-1β, IL-6, IL-8, IL-10, IL-12/23p40, IL-17, IL-21, TNF-α, G-CSF, GM-CSF, PCT	35 febrile neutropenic pediatric oncology patients, Brazil	IL-6 > 170 pg/mL: Sens. 69%, Spec. 95%, PPV 90%, NPV 84%; IL-8 > 240 pg/mL: Sens. 69%, Spec. 100%, PPV 100%, NPV 85%; IL-10 > 6 pg/mL: Sens. 69%, Spec. 86%, PPV 75%, NPV 83%; PCT > 180 pg/mL: Sens. 80%, Spec. 68%, PPV 50%, NPV 89%	IL-6, IL-8, IL-10, and PCT were significantly elevated on day 1 in patients who developed sepsis. IL-8 and IL-10 decreased after day 2. IL-12/23p40 and IL-17 were higher in non-septic patients. Filgrastim significantly increased IL-6, IL-8, IL-10 levels, but had minimal effect on diagnostic performance.	Deficiency in IL-23/IL-17 axis and IL-21 expression in septic patients may reflect immunosuppression; not quantitatively analyzed as prognostic markers.	IL-12/23p40 and IL-17 were significantly higher in non-septic patients, suggesting their role in mucosal immunity and bacterial translocation prevention.	Combination of markers (e.g., IL-8 > 240 + high risk, or IL-6 > 50 + PCT > 100) improved stratification performance.
29	Gupta (2020) [33]	IL-6, CRP	32 episodes of febrile neutropenia in 25 pediatric oncology patients in South India; prospective observational study	IL-6 median values: Gram-negative 169, Gram-positive 17.5, sterile 52 pg/mL; CRP median: Gram-negative 60.7, Gram-positive 85.5, sterile 44.2 mg/L. IL-6 levels ranged from 8 to 5000 pg/mL; CRP: 0.66–288 mg/L.	IL-6 was elevated in all episodes and significantly higher in Gram-negative bacteremia (*p* = 0.017); CRP did not show such discriminatory power (*p* = 0.796).	All 4 deaths had IL-6 > 100 pg/mL; MDI had the highest IL-6 and worst outcomes (3/7 deaths, prolonged neutropenia in 85.8%).	IL-6 was a better predictor of Gram-negative sepsis than CRP.	IL-6 may help identify high-risk Gram-negative infections; CRP remains useful when IL-6 is unavailable.
30	Nijhuis (2005) [34]	IL-8	196 febrile neutropenic episodes in 128 cancer patients (children and adults), Netherlands; prospective, interventional single-center study	IL-8 cutoff: 60 ng/L. Low-risk: IL-8 ≤ 60 ng/L and no abnormal vital signs. Median IL-8 levels: low-risk 23 (7–45), medium-risk 120 (29–4927), high-risk 92 (13–9384) ng/L.	IL-8-based model (with vitals) correctly identified low-risk patients. Sensitivity: 100%, specificity: 21%, NPV: 100%, PPV: 13%. No bacteremia or failures in low-risk group.	not applicable	IL-8 levels at admission helped stratify risk of bacterial infection; no bacteremia occurred in patients with IL-8 ≤ 60 ng/L and no signs of sepsis.	IL-8 combined with clinical parameters allowed identification of patients safe for early discharge and without need for antibiotics, reducing costs and hospitalization.
31	Xia (2016) [35]	IL-2, IL-4, IL-6, IL-10, TNF-α, IFN-γ, PCT, CRP	2819 febrile episodes in 828 pediatric hematology/oncology patients, China; retrospective study	IL-6 ≥ 500 pg/mL: Sens. 54.4%, Spec. 92.5%; IL-10 ≥ 100 pg/mL: Sens. 47.1%, Spec. 94.9%; PCT ≥ 2 ng/mL: Sens. 29.4%, Spec. 95% (severe infection). TNF-α ≥ 50 pg/mL: Sens. 18.1%, IFN-γ ≥ 25 pg/mL: Sens. 25.6%, IL-6/IL-10 positivity in Gram-negative bacteremia: 96.8%/90.3% vs. TNF-α/IFN-γ: 18.1%/25.6%.	IL-6 and IL-10 were superior to TNF-α/IFN-γ in detecting bacteremia/severe infection. TNF-α/IFN-γ had low sensitivity (<30%) but correlated with inflammation severity.	IL-6/IL-10 levels correlated with infection severity and septic shock risk; TNF-α/IFN-γ had limited prognostic utility.	IL-6/IL-10 had highest accuracy for severe infection (AUC 0.875/0.839), but moderate accuracy for Gram-negative bacteremia (AUC 0.686/0.747)	IL-6/IL-10 thresholds (≥500/≥100 pg/mL) identify high-risk patients; TNF-α/IFN-γ are not recommended for risk stratification.
32	Hemming (2017) [36]	PCT	48 episodes of febrile neutropenia in 27 pediatric cancer patients, UK; prospective cohort study	PCT > 2 ng/dL: LR = 26 [95% CI 3.5–190]; PCT < 0.5: LR = 0.32; intermediate (0.5–2): LR = 0.39. No correlation with neutrophil count (*r* = −0.08).	High PCT on admission significantly associated with severe infection; clinical decision rules alone frequently overstated risk.	PCT > 2 ng/dL correlated with severe outcomes, but data insufficient to assess multiple-day predictive value.	not applicable	No definitive benefit in using PCT in pediatric FN
33	Nonkulovski (2020) [37]	PCT	20 pediatric hemato-oncology patients with febrile neutropenia and sepsis; North Macedonia; retrospective-prospective study	All 20 patients had PCT ≥ 2 ng/mL within 24 h of admission. In septic shock: PCT > 10 ng/mL. PCT decreased after 3–5 days of antibiotic therapy; further drop by 6–14 days.	Elevated PCT confirmed sepsis early; levels decreased with treatment. Used to guide initiation and discontinuation of antibiotics.	PCT > 10 ng/mL associated with septic shock and fatal outcomes; dynamic PCT decline reflected response to treatment.	not applicable	PCT used to tailor duration and intensity of antibiotic therapy; supports rational antibiotic use and resistance prevention.
34	Zareifar (2020) [38]	PCT, CRP, ESR	107 pediatric cancer patients with febrile neutropenia; cross-sectional study, Iran	PCT ≥ 0.70 ng/mL: Sens. 76%, Spec. 74.4%, PPV 47.5%, NPV 91%; AUC = 0.74 (95% CI: 0.61–0.87); *p* < 0.001	PCT levels significantly higher in patients with positive blood cultures (median 2.17 vs. 0.32 ng/mL, *p* < 0.001); superior to ESR and comparable to CRP.	not applicable	PCT was significantly higher in culture-positive cases; suggests value in detecting infection, especially bloodstream infection.	High NPV suggests PCT may help rule out serious infection in febrile neutropenic patients; useful to guide clinical management.
35	Özdemir (2019) [39]	PCT, CRP, Presepsin, sTREM-1	47 episodes of febrile neutropenia in 30 pediatric oncology patients (vs. 27 controls); Turkey	PCT (cutoffs): Day 1 = 0.5 ng/mL (Sens. 61.5%, Spec. 89.4%, AUC =0.722), Day 2 = 0.25 ng/mL (Sens. 84.6%, Spec. 55.9%, AUC = 691), Day 7 = 0.5 ng/mL (Sens. 53.8%, Spec. 85.3%). CRP (cutoffs): Day 1 = 2.5 mg/dL, Day 2 = 4 mg/dL, Day 7 = 7 mg/dL. Presepsin and sTREM-1 had no significant diagnostic performance.	PCT and CRP were significantly higher in culture-positive vs. culture-negative episodes. PRE-SEP and sTREM-1 were elevated but lacked discriminatory power.	PCT and CRP levels on days 2 and 7 were higher in patients with clinical signs of sepsis; sTREM-1 levels were numerically higher in sepsis but not statistically significant.	Presepsin and sTREM-1 did not distinguish culture-positive from negative; PCT and CRP had moderate diagnostic accuracy.	PCT and CRP within 24 h useful for guiding therapy; Presepsin and sTREM-1 not recommended for risk stratification in this context.
36	Purkayastha (2016) [40]	PCT, CRP	82 febrile neutropenia episodes in pediatric oncology patients; India; prospective observational study	PCT ≥ 0.25 ng/mL: Sens. 73.3%, Spec. 29.4%, PPV 18.6%, NPV 83.3%; CRP ≥ 110 mg/L: Sens. 13.3%, Spec. 77.2%, PPV 13.3%, NPV 77.2%	PCT was more sensitive than CRP, particularly in pulmonary infections. However, specificity was low, and PCT values showed wide variability. CRP had higher specificity but poor sensitivity.	not applicable	PCT levels were higher in pulmonary than extrapulmonary infections; CRP was less useful in distinguishing etiologies.	Authors emphasize that PCT should be used routinely. Its low specificity and high variability limit its standalone value. Further validation is needed.
37	Baraka (2018) [41]	Presepsin, PCT, CRP	60 pediatric FN patients with hematological malignancies (ALL, AML, NHL, HD); Egypt; case-control study	Presepsin ≥ 1014 pg/mL (bacteremia): Sens. 100%, Spec. 85.7%, AUC = 0.95; ≥ 951 pg/mL (bacteremia + CPI): Sens. 93.8%, Spec. 100%, AUC = 0.996. PCT ≥ 2.86 ng/mL: Sens. 100%, Spec. 81%, AUC = 0.92. CRP ≥ 105 mg/L: Sens. 77.8%, Spec. 66.7%, AUC = 0.75.	Presepsin showed excellent accuracy (AUC 0.996) for bacteremia + CPI, outperforming CRP but comparable to PCT (AUC 0.92). PCT retained 100% sensitivity for bacteremia. Presepsin levels were higher in bacteremia vs. CPI and FUO; positively correlated with PCT and CRP, negatively with ANC.	not applicable	Presepsin levels did not significantly differ between Gram-positive and Gram-negative infections.	Authors recommend combining presepsin with CRP to improve diagnostic sensitivity. PCT remains valuable due to its high sensitivity. Study limited by small sample size and lack of external validation.
38	El-Maghraby (2007) [42]	CRP, IL-8, MCP-1-a	85 febrile neutropenia episodes in 76 pediatric patients with hematologic malignancies (ALL, ANLL, NHL); Egypt; prospective study	CRP ≥ 90 mg/L: Sens. 69.5%, Spec. 73.1%, PPV 85.4%, NPV 51.4%. IL-8 ≥ 62 pg/mL: Sens. 71.2%, Spec. 76.5%, PPV 87.5%, NPV 54.1%. MCP-1-a ≥ 350 pg/mL: Sens. 64.4%, Spec. 92.3%, PPV 95%, NPV 53.3%.	CRP, IL-8 and MCP-1-a were significantly elevated in infected patients (*p* < 0.001). CRP ≥ 90 mg/L had highest sensitivity (100%) for bacteremia; MCP-1-a had the highest specificity. IL-8 and MCP-1-a had limited sensitivity for bacteremia.	not applicable	CRP levels were higher in bacteremia (especially Gram-negative and S. aureus); MCP-1-a and IL-8 levels did not differ significantly between Gram(+) and Gram(−) infections.	CRP showed superior sensitivity (100%) for bacteremia at ≥90 mg/L, while MCP-1-a had the highest specificity (92.3%). Combining markers improved accuracy: 78% of infected patients had ≥2 elevated markers vs. 16% in non-infectious cases. Authors recommend CRP as the primary marker and highlight study limitations.
39	Cerasi (2023) [43]	Presepsin, CRP, PCT, IL-6	41 pediatric patients with hematological/oncological diseases experiencing 50 febrile neutropenia episodes; 100 healthy controls; Italy	Presepsin T0 (at fever onset, before antibiotics): cutoff 410 pg/mL (AUC = 0.508; Se = 0.53, Sp = 0.55, PPV = 0.84, NPV = 0.22); T1 (48 h later): cutoff 213 pg/mL (AUC = 0.423; Se = 0.90, Sp = 0.50, PPV = 0.90, NPV = 0.50). IL-6 T0: AUC = 0.748; PCT T0: AUC = 0.793	Presepsin was not useful for detecting bacteremia (low sensitivity/specificity, AUC < 0.6). PCT and IL-6 showed better accuracy.	Presepsin T1 ≥ 923 pg/mL predicted unfavorable outcome: Se = 0.75, Sp = 0.89, PPV = 0.37, NPV = 0.98, AUC = 0.792. IL-6 and CRP at T1 were also significant predictors.	Presepsin was ineffective in differentiating Gram+ from Gram− bacteremia; IL-6 was more useful at T0 for distinguishing bacterial infections.	Authors suggest presepsin may help rule out poor outcome due to its high NPV at T1; recommend combining biomarkers (IL-6, PCT, presepsin) for early risk stratification.
40	Arıkan (2021) [44]	Presepsin, proADM, CRP, PCT	47 febrile neutropenia episodes in 39 pediatric patients with hematologic malignancies; 40 healthy controls; Turkey	Presepsin day 7 cutoff 750 pg/mL (AUC = 0.74; Se = 85%, Sp = 64%); PCT day 1 cutoff 0.36 ng/mL (AUC = 0.74; Se = 82%, Sp = 62%); proADM day 1 cutoff 57.95 pg/mL (AUC = 0.78; Se = 98%, Sp = 75%)	Presepsin and PCT effective in detecting bacteremia in FN; proADM most valuable early marker with highest sensitivity (98%); notably, presepsin levels were higher in healthy controls than FN patients due to neutropenia effect	Study focused on diagnostic accuracy for bacteremia detection, not prognostic outcomes; proADM showed highest diagnostic performance for early bacteremia detection	PCT significantly higher in Gram− vs. Gram+ bacteremia (*p* = 0.01 for day 1, *p* = 0.008 for day 2); CRP higher in Gram− vs. Gram+ only on day 2 (*p* = 0.02, NS on day 1); presepsin not significantly different between bacterial subtypes	Multivariate analysis showed that combining presepsin day 1 with PCT1/CRP1 was superior to individual markers for early bacteremia prediction in FN episodes
41	Antari (2023) [45]	PSP; MR-proADM, CRP	70 children (<18 years) with hematological malignancies (ALL, AML, NHL); 70 episodes of febrile neutropenia; Greece	PSP on day 1: median 179 ng/mL (sepsis) vs. 80 ng/mL (no sepsis), *p* < 0.00001. MR-proADM: 0.559 vs. 0.196 nmol/L, *p* = 0.02. CRP: 7.08 vs. 3.04 mg/dL, *p* = 0.06. Diagnostic performance: PSP–Se = 84%, Sp = 82%; MR-proADM–Se = 74%, Sp = 70%; CRP–Se = 88%, Sp = 57%	PSP and MR-proADM were effective in early sepsis detection; PSP showed the best overall diagnostic accuracy	Prognostic evaluation not detailed; outcomes included 4% 28-day mortality and 10% ICU admission	PSP and MR-proADM levels were significantly higher in bloodstream infections (*p* = 0.03 and *p* = 0.04, respectively)	Not explicitly discussed, but PSP considered promising for early identification of high-risk cases (e.g., ICU need)
42	Agnello (2020) [46]	Presepsin, MR-ProADM	37 febrile neutropenia episodes in 26 pediatric oncology patients; single-center study; Italy	Presepsin and mr-proADM were elevated at admission (T0) and decreased by day 5 (T2). Presepsin differed significantly between bacteremia and FUO groups at T1 only (median 416 vs. 271 pg/mL, *p* = 0.021). Diagnostic accuracy was low: Presepsin AUC = 0.58; mr-proADM AUC = 0.62	Both biomarkers showed poor utility in identifying blood culture positivity; low diagnostic value in distinguishing infection etiology	Presepsin and mr-proADM at T0 were significant predictors of hospital length of stay (PSP: *p* = 0.00007; mr-proADM: *p* = 0.0038), but not of fever duration	Only PSP showed significant differences between bacteremia and FUO at T1; mr-proADM did not differ significantly at any timepoint	Elevated Presepsin and mr-proADM were associated with longer hospitalization, suggesting limited prognostic value; no specific risk stratification model proposed
43	Ragab (2025) [47]	CRP, PCT, MR-ProADM	137 pediatric patients with chemotherapy-induced febrile neutropenia; Egypt	MR-ProADM cutoff: 489 pg/mL (AUC = 0.964, Se = 90.5%, Sp = 82.6%, PPV = 87.9%, NPV = 88.2%). CRP and MR-ProADM levels significantly higher in bacterial infection (CRP: 114.2 ± 12.16 vs. 68.9 ± 10.56 mg/dL; MR-ProADM: 703.65 ± 100.3 vs. 492.04 ± 124.9 pg/mL; *p* < 0.001)	MR-ProADM, CRP, and PCT were all elevated in bacterial infections; MR-ProADM showed highest diagnostic accuracy for infection prediction	Higher levels of MR-ProADM, CRP, and PCT correlated with longer FN duration, hospital stay, and mortality. MR-ProADM showed strongest correlation with hospitalization length (*r* = 0.838)	MR-ProADM and CRP significantly higher in bacteremia cases than non-bacteremia	MR-ProADM suggested as a reliable diagnostic and prognostic biomarker in FN; no formal algorithm proposed but strong potential indicated
44	Fawzi (2019) [48]	CRP, MR-ProADM	100 pediatric patients (1–15 years) with hematologic malignancies and severe neutropenia; Egypt	MR-proADM day 1 cutoff: 2.4 nmol/L (AUC = 0.939, Se = 91.6%, Sp = 85.1%, PPV = 83.3%, NPV = 92.4); CRP day 1 cutoff: 94 mg/L (AUC = 0.509, Se = 52.6%, Sp = 70.9%). MR-proADM levels significantly higher in BS vs PUO on both days 1 and 2	MR-proADM significantly outperformed CRP in early detection of bacteremia/sepsis versus PUO; CRP was a poor diagnostic marker (AUC ~0.51)	Prognostic value not assessed due to short follow-up; no data on outcomes beyond diagnostic performance	MR-proADM effectively differentiated BS from PUO from day 1; CRP levels showed no significant differences	MR-proADM considered a reliable early biomarker to aid in sepsis diagnosis in febrile neutropenic children with hematologic malignancies
45	Susanto (2022) [49]	MR-ProADM	60 pediatric patients (1–18 years) with cancer-related chemotherapy (30 with sepsis, 30 without sepsis); Indonesia	MR-ProADM cutoff: 2.88 nmol/L; AUC = 0.707; Se = 60.0%, Sp = 56.67%, PPV = 58.06%, NPV = 58.62%, LR+ = 1.38, LR− = 0.71, diagnostic accuracy = 59.33%. Unexpectedly, median MR-ProADM levels were higher in the non-sepsis group (2.51 vs. 0.194 nmol/L), *p* = 0.006	MR-ProADM showed moderate overall diagnostic accuracy (AUC = 0.707) for sepsis in pediatric cancer patients, but limited sensitivity and specificity	Not evaluated; the study focused only on diagnostic performance	MR-ProADM levels were paradoxically higher in non-sepsis group than in sepsis group, suggesting inverse association with clinical diagnosis	Authors concluded that MR-ProADM alone has limited diagnostic value; may be more effective when used alongside clinical criteria or in combination with other biomarkers
46	Demirkaya (2015) [50]	MR-ProADM CRP, PCT	50 febrile neutropenia episodes in 37 pediatric cancer patients (leukemia, lymphoma, solid tumors); Turkey	ADM levels on day 3 were significantly higher in MDI group vs CDI and FUO [MDI: 191.7 (32.1–695.4), CDI: 42.8 (21.2–110.1), FUO: 46.0 (23.3–91.3), sepsis: 58.7 (19.7–100.1) pg/mL]; no cutoff provided	ADM could identify microbiologically documented infections on day 3 but was not helpful in differentiating sepsis; diagnostic utility limited compared to PCT	ADM levels were not associated with mortality or infection severity. In contrast, PCT was significantly elevated in deceased patients and in severe infections	ADM levels significantly higher in MDI vs FUO/CDI but not in sepsis; CRP showed no significant differentiation across groups; PCT most useful for identifying infection severity	Due to ADM’s rapid clearance, authors suggest limited standalone utility and recommend future studies using proADM for improved stability and stratification
